# *Phytophthora infestans* RXLR effector SFI5 requires association with calmodulin for PTI/MTI suppressing activity

**DOI:** 10.1111/nph.15250

**Published:** 2018-06-22

**Authors:** Xiangzi Zheng, Nadine Wagener, Hazel McLellan, Petra C. Boevink, Chenlei Hua, Paul R. J. Birch, Frédéric Brunner

**Affiliations:** ^1^ Department of Biochemistry Centre for Plant Molecular Biology Eberhard Karls University Auf der Morgenstelle 32 D‐72076 Tübingen Germany; ^2^ Center for Molecular Cell and Systems Biology College of Life Sciences Fujian Agriculture and Forestry University Fuzhou 350002 China; ^3^ Division of Plant Sciences University of Dundee (at James Hutton Institute) Errol Rd Invergowrie, Dundee DD2 5DA UK; ^4^ Cell and Molecular Sciences The James Hutton Institute Errol Rd Invergowrie, Dundee DD2 5DA UK; ^5^ PlantResponse Biotech, S.L. Centre for Plant Biotechnology and Genomics (CBGP) Campus de Montegancedo 28223 Pozuelo de Alarcón, Madrid Spain

**Keywords:** calcium, calmodulin, MAMP‐triggered immunity (MTI), RXLR effector, SFI5, tomato protoplast, virulence

## Abstract

- Pathogens secrete effector proteins to interfere with plant innate immunity, in which Ca^2+^/calmodulin (CaM) signalling plays key roles. Thus far, few effectors have been identified that directly interact with CaM for defence suppression. Here, we report that SFI5, an RXLR effector from *Phytophthora infestans*, suppresses microbe‐associated molecular pattern (MAMP)‐triggered immunity (MTI) by interacting with host CaMs.
- We predicted the CaM‐binding site in SFI5 using *in silico* analysis. The interaction between SFI5 and CaM was tested by both *in vitro* and *in vivo* assays. MTI suppression by SFI5 and truncated variants were performed in a tomato protoplast system.
- We found that both the predicted CaM‐binding site and the full‐length SFI5 protein interact with CaM in the presence of Ca^2+^. MTI responses, such as *FRK1* upregulation, reactive oxygen species accumulation, and mitogen‐activated protein kinase activation were suppressed by truncated SFI5 proteins containing the C‐terminal CaM‐binding site but not by those without it. The plasma membrane localization of SFI5 and its ability to enhance infection were also perturbed by loss of the CaM‐binding site.
- We conclude that CaM‐binding is required for localization and activity of SFI5. We propose that SFI5 suppresses plant immunity by interfering with immune signalling components after activation by CaMs.

## Introduction

Plants rely on a multi‐layered immune system to combat potential pathogens. One important layer of inducible plant immune responses is the recognition by cell surface‐resident pattern recognition receptors (PRRs) of so‐called microbe‐associated molecular patterns (MAMPs), which are highly conserved molecules or structural components derived from microbes and indispensable for microbial fitness or lifestyle (Medzhitov & Janeway, 1997; Nürnberger & Brunner, 2002; Nürnberger *et al*., 2004; Ausubel, 2005; Boller & Felix, 2009; Macho & Zipfel, 2014). The signalling responses induced by PRR‐mediated perception of MAMPs is termed MAMP‐triggered immunity (MTI), which is accompanied by rapid Ca^2+^ influxes, accumulation of reactive oxygen species (ROS), activation of mitogen‐activated protein kinases (MAPKs), upregulation of immunity‐associated gene expression, synthesis of anti‐microbial proteins and callose deposition (Apel & Hirt, 2004; Macho & Zipfel, 2014; Bigeard *et al*., 2015; Lee *et al*., 2015).

One of the key mediators in plant immune responses is Ca^2+^/calmodulin (CaM) (Lecourieux *et al*., 2006; Cheval *et al*., 2013). CaM is a major Ca^2+^ sensor in eukaryotes and, after conformational change induced by Ca^2+^ binding to EF‐hand motifs, interacts with and regulates the function of diverse target proteins (McCormack *et al*., 2005). These include membrane receptors, protein kinases and transcription factors that are involved in plant development but also in plant immunity and adaptation to other stress conditions (Zhang *et al*., 2009; Reddy *et al*., 2011; Cho *et al*., 2016).

Microbes that can colonize plants have evolved multiple effectors that either prevent perception of MAMPs by membrane‐bound PRRs or interfere with cellular components of MTI signalling pathways (Boller & He, 2009; Bozkurt *et al*., 2012; Dou & Zhou, 2012; Giraldo & Valent, 2013; Doehlemann *et al*., 2014). The latter mainly involves effectors that translocate into host cells. For example, the bacterial pathogen *Pseudomonas syringae* secretes many effectors into plant cells using the type III secretion system, targeting various proteins to suppress MTI (Block & Alfano, 2011; Dou & Zhou, 2012; Fraiture & Brunner, 2014). Recently, one effector protein, HopE1, was shown to interact directly with CaM for MTI suppression (Guo *et al*., 2016). Oomycete pathogens, including downy mildews and *Phytophthora* species, secrete hundreds of candidate effectors that contain the RXLR motif shortly after the N‐terminal signal peptide (Tyler *et al*., 2006; Jiang *et al*., 2008; Haas *et al*., 2009), which is required for delivery into host cells (Whisson *et al*., 2007, 2016; Dou *et al*., 2008). Some of these effectors have been shown to target MTI components and suppress plant immunity (King *et al*., 2014; Whisson *et al*., 2016).

*Phytophthora infestans* is the causal agent of tomato and potato late blight disease, but it can also infect the model solanaceous plant *Nicotiana benthamiana* (Zheng *et al*., 2014; Whisson *et al*., 2016). Many RXLR effectors in *P. infestans* and other well‐known oomycete pathogens such as *Phytophthora sojae* and *Hyaloperonospora arabidopsidis* have been shown to suppress MTI at different steps of the signalling pathway, through different mechanisms and in different subcellular compartments. *P. infestans* effector AVR1 interacts with host exocyst component Sec5 to suppress CRN2‐induced cell death and PR1 secretion (Du *et al*., 2015). AVR3a represses the programmed cell death induced by the elicitor INF1 by targeting and stabilizing the host E3 ligase CMPG1 (Bos *et al*., 2006, 2010; Gilroy *et al*., 2011). PITG_03192 localizes to the host endoplasmic reticulum (ER), where it targets two NAC transcription factors, NTP1 and NTP2, and prevents their re‐localization into the nucleus upon MAMP application (McLellan *et al*., 2013). Pi04314 associates with different isoforms of host protein phosphatase type 1c (PP1c) and causes their re‐localization within the nucleus without affecting their biochemical activity. The PP1c isoforms were proposed to be susceptibility factors, utilized by Pi04314 to promote disease development by attenuating salicylic acid (SA) and jasmonate (JA) signalling (Boevink *et al*., 2016). PexRD2 interacts with host MAPK signalling to perturb both MTI and ETI (King *et al*., 2014). In *P. sojae*, Avr3b contains a Nudix hydrolase motif in the C‐terminal part of the effector domain and displays ADP‐ribose/NADH pyrophosphorylase enzymatic activity, which is dependent on the interaction with the plant cyclophilin CYP1 through a putative Glycine‐Proline motif (Dong *et al*., 2011; Kong *et al*., 2015). PsAvh23 suppresses defence gene expression by interacting with the ADA2 subunit of the histone acetyltransferase complex (Kong *et al*., 2017). In *H. arabidopsidis*, the nuclear‐localized effector, HaRxL44, was shown to associate with Mediator subunit 19a (MED19a), leading to proteasome‐dependent degradation of MED19a and the activation of jasmonate/ethylene (JA/ET)‐signalling to antagonize SA‐signalling and the activation of SA‐responsive genes (Caillaud *et al*., 2013).

In our previous study, we identified a range of *P. infestans* RXLR effectors, named Suppressor of early Flg22‐induced Immune response (SFI), that perturb the earliest signalling events of MTI in tomato protoplasts (Zheng *et al*., 2014). One of these, namely SFI5, localizes at the host plasma membrane, blocks the MAMP‐triggered activation of the MAP kinase cascade and facilitates *P. infestans* infection in *N. benthamiana* leaves. The mechanism underlying the mode of action of SFI5 in manipulating host immunity was not investigated.

In this study, we show that SFI5 contains a CaM‐binding motif at its C‐terminus. We demonstrate Ca^2+^‐dependent association between SFI5 and CaMs in both *in vitro* and *in vivo* assays. We provide strong correlative evidence that CaM‐association is required for the plasma membrane location and effector activity of SFI5 in tomato protoplasts and *N. benthamiana* leaves. We report for the first time that an effector association with CaM plays a critical role in suppression of immunity in the interactions between plants and filamentous pathogens.

## Materials and Methods

### Bioinformatics

The sequence of *Phytophthora infestans* SFI5 (PITG_13628) was analysed for CaM‐binding domains and canonical motifs according to the method described by Yap *et al*. (2000) and Mruk *et al*. (2014). Calmodulin sequences from Arabidopsis (*Arabidopsis thaliana*) and tomato (*Solanum lycopersicum*) were obtained from GenBank according to the descriptions of Day *et al*. (2002) and Zhao *et al*. (2013). Sequence alignment was performed by ClustalW program (Chenna *et al*., 2003).

### Microbe and plant growth conditions

*Escherichia coli* DH5α and Rosetta™(DE3) (Merck, Germany) were routinely grown in Luria Bertani (LB) media with appropriate antibiotics at 37°C. *Phytophthora infestans* strain 88069 (Pieterse & Davidse, 1990) was grown on Rye Sucrose Agar at 19°C. *Nicotiana benthamiana* Domin (personal laboratory stock) and *Solanum lycopersicum* L. cv Moneymaker (Thompson & Morgan, Ipswich, Suffolk, UK) were grown under controlled conditions in a glasshouse as described previously (Zheng *et al*., 2014).

### Gene cloning and constructs

Calmodulin genes were cloned by PCR amplification from tomato cDNAs with primer pairs shown in Supporting Information Table S1. The PCR amplicons were cloned using the Gateway^®^ cloning recombination technology into the entry vector pDONR201 (Thermo Fisher Scientific, Darmstadt, Germany) and, subsequently, recombined into the expression vectors p2HAGW7 (N‐terminal HA‐tag) (Zheng *et al*., 2014) and p2FGW7 (C‐terminal GFP‐tag) or pB7WGF2 (N‐terminal GFP‐tag) (VIB, Belgium; Karimi *et al*., 2005), or pDEST15 (N‐terminal GST‐tag) and pDEST17 (N‐terminal His‐tag) for protein expression in *E. coli* (Thermo Fisher Scientific). The *SFI5* gene without the region encoding the signal peptide (28–241 aa) was amplified by PCR, digested with *Xmn*I and *Sal*I restriction endonucleases and ligated into the vector pMAL‐p5x (N‐terminal MBP‐tag) (New England Biolabs, Frankfurt, Germany). Positive clones were confirmed by sequencing.

Truncated *SFI5* genes without fragments encoding N‐ or C‐terminal domains were amplified by PCR using specific primers (Table S1), cloned into the entry vector pDONR201 (Thermo Fisher Scientific) and subsequently recombined into the expression vectors p2HAGW7 and p2FGW7 (Life Technologies, Darmstadt, Germany). Site‐directed mutagenesis was performed following the instruction manual of the QuikChange^®^ II XL Site‐Directed Mutagenesis Kit (Agilent Technologies, Waldbronn, Germany) using the p2HAGW7‐SFI5 63–241 aa or p2FGW7‐SFI5 63–241 aa plasmid constructs as PCR template. Primers used for mutagenesis are listed in Table S1. All constructs were verified by sequencing.

### *In vitro* pull‐down assay

pDEST17 vector expressing His‐SlCaM6 and pMAL‐p5x vector expressing MBP‐SFI5 (28–241 aa) were transformed into Rosetta™(DE3) competent cells, respectively. Positive colonies were cultivated in LB medium at 37°C with 200 rpm shaking until the culture reached an OD_600_ of 0.6, followed by addition of 0.5 mM isopropyl β‐d‐thiogalactopyranoside (IPTG) for protein induction. Upon induction, bacterial cultures were transferred to 28°C with 200 rpm shaking for 2 h before harvesting. His‐ and MBP‐fusion proteins were purified by HisTrap (HisTrap FF Crude; GE Healthcare, Freiburg, Germany) and MBPTrap (MBPTrap HP; GE Healthcare) columns, respectively, following the user manuals. For pull‐down assay, the same amount (5 mmol) of His‐SlCaM6 and MBP‐SFI5 were mixed together with 5 mM CaCl_2_ or 5 mM EGTA in 500 μl PBS buffer and incubated at 4°C for 1 h. Both protein mixtures were then incubated with 10 μl Ni‐NTA resin at 4°C for 30 min, followed by the removal of the supernatant and extensive wash (eight times) with wash buffer (250 mM This‐HCl, 500 mM NaCl, 25% (v/v) glycerol, 5 mM β‐mercaptoethanol, and 10 mM imidazole. pH 8.0). The binding proteins were eluted with 1 M imidazole and mixed with SDS sample buffer for SDS‐PAGE. Purified His‐SlCaM6 and MBP‐SFI5 proteins also were loaded for SDS‐PAGE as controls.

### Transfection of tomato protoplasts

Tomato mesophyll protoplasts were isolated and transfected as described previously (Fraiture *et al*., 2014; Zheng *et al*., 2014).

### Immunoprecipitation and mass spectrometry

The transformed protoplasts were harvested by centrifugation at 100 ***g*** for 1 min and the pellet was re‐suspended in 1 ml of immunoprecipitation (IP) buffer containing 50 mM HEPES (pH 7.4), 150 mM NaCl, 0.1% Triton X‐100, 1 mM EDTA, 1 mM DTT, 1× phosphatase inhibitor cocktail (PhosphoSTOP, Roche) and 1× protease inhibitor cocktail. Total protein was released by sonication and the cell debris was removed by centrifugation. The HA‐tagged proteins were immunoprecipitated from lysates by incubation with 20 μl of anti‐HA antibody‐coupled beads (anti‐HA affinity matrix, Roche) for 3–6 h while gently shaking at 4°C. Afterwards, the beads were washed three times with 1 ml of washing buffer (50 mM HEPES pH 7.4, 150 mM NaCl, 0.2% Triton X‐100, 1× phosphatase inhibitor cocktail and 1× protease inhibitor cocktail). For elution, 50 μl SDS loading buffer without DTT was added to the beads, followed by boiling at 95°C for 10 min. The immunoprecipitated proteins were then further analysed by immunoblotting or LC‐MS/MS (tandem).

### SDS‐PAGE and immunoblotting

Proteins were separated by 13.5% SDS‐PAGE, blotted and incubated with appropriate antibodies (Zheng *et al*., 2014). The antibody dilutions were: anti‐phospho‐p44/42 MAPK antibody (Cell Signalling Technology Europe BV, Leiden, Netherlands), 1 : 1000 in 5% BSA‐TBST; anti‐GFP (Acris), 1 : 5000 in 5% BSA‐TBST; anti‐HA (Sigma), 1 : 1000 in 5% BSA‐TBST; anti‐GST (Sigma), 1 : 7000 in 5% BSA‐TBST; anti‐MBP (Cell Signalling Technology), 1 : 10 000 in 5% BSA‐TBST; anti‐His (Abcam, Cambridge, UK), 1 : 5000 in 5% BSA‐TBST; anti‐rabbit IgG alkaline phosphatase (Sigma), 1 : 3000 in TBST; anti‐goat IgG alkaline phosphatase (Sigma), 1 : 3000 in TBST; anti‐mouse IgG alkaline phosphatase (Sigma), 1 : 3000 in TBST; anti‐goat IgG horseradish peroxidase (Sigma), 1 : 10 000 in TBST; anti‐mouse IgG horseradish peroxidase (Sigma), 1 : 10 000 in TBST. Immunodetection was performed with a NBT/BCIP solution (AppliChem, Darmstadt, Germany) or with the ECL Western blotting system (GE Healthcare).

### Tris‐glycine native‐PAGE

pDEST15 vector expressing GST‐SlCaM6 was transformed into Rosetta™(DE3) competent cells, and the fusion protein was induced and purified with GSTrap column (GSTrap FF column, GE Healthcare) following the user manual. Native polyacrylamide gel electrophoresis (Native‐PAGE) was carried out as described previously (Niepmann & Zheng, 2006; Arndt *et al*., 2012). Equal volumes of 133 μM synthetic peptide and 50 μM GST‐ or His‐ SlCaM6 were mixed and incubated for 1 h at 4°C. Peptide–protein interactions were analysed on 12% tris‐glycine native gels (30 mM Tris‐HCl pH 7.5, 190 mM glycine, 5 mM DTT). NativeMark Unstained Protein Standard (Life Technologies) was used as a protein marker for the analysis. The electrophoresis was performed with the Mini‐PROTEAN Tetra Cell apparatus (BioRad) in native running buffer (25 mM Tris base, 192 mM Glycine) at a low current (10 mA) at 4°C. The proteins on the gel were visualized by Coomassie blue staining (Blakesley & Boezi, 1977).

### 1‐Anilinonaphthalene‐8‐sulfonate assay

1‐Anilinonaphthalene‐8‐sulfonate (ANS) (Sigma) was dissolved in ethanol at a stock concentration of 10 mM. The assays were performed as described by Chinpongpanich *et al*. (2011) with minor modifications. ANS (100 μM) and 1 μM purified recombinant GST‐SlCaM6 were incubated in the reaction buffer containing 20 mM Tris‐HCl (pH 7.5), 100 mM NaCl, and 1 mM CaCl_2_ for 15 min before addition of 0–100 μM synthetic peptide. Fluorescence was measured in a 96‐well plate reader (MWG) with λ_ex_ = 360 nm and λ_em_ = 460 nm.

### Luciferase reporter gene assays

Luciferase reporter gene assay was conducted following the method of Zheng *et al*. (2014). Protoplasts were treated with/without flg22 to a final concentration of 500 nM. The luminescence reflecting the luciferase activity was measured at different time‐points using a Mithras LB 940 luminometer (Berthold, Bad Wildbad, Germany). Luciferase activity (+/−flg22) was normalized by measuring β‐glucuronidase (GUS) activity upon lysis with an equal volume of CCLR solution and incubation of 10 μl protoplast extract with 90 μl MUG substrate.

### MAP kinase activation assay

The MAP kinase activation was detected as described previously (Zheng *et al*., 2014). Two hundred microliters of transformed tomato protoplasts untreated or treated with 500 nM flg22 for 0 and 20 min were harvested for immunodetection by anti‐phospho‐p44/42 MAPK antibody.

### Oxidative burst assay

Tomato protoplasts were used to measure reactive oxygen species (ROS) production using a luminol‐based assay (Halter *et al*., 2014). One hundred to 200 μl of transformed protoplast samples were incubated in W1 buffer without MES (0.5M mannitol, 20 mM KCl) at 20–22°C in the dark for 6–8 h. Before measurement, protoplasts were harvested by centrifugation at 100 ***g*** for 1 min and resuspended in 100 μl W5 buffer (18.4 g l^−1^ CaCl_2_ × 2H_2_O, 1.0 g l^−1^ glucose, 9.0 g l^−1^ NaCl, 0.4 g l^−1^ KCl) containing 200 μM luminol L‐012 (Wako Chemicals) and 20 μg ml^−1^ horseradish peroxidase and incubated for an additional 30 min in the dark. After treatment with 500 nM flg22, luminescence was recorded for 30 min using a Mithras LB 940 multiplate reader (Berthold).

### Infection assay

*Phytophthora infestans* infection assay was performed as described previously (Zheng *et al*., 2014). *Agrobacterium tumefaciens* carrying GFP‐SFI5 deletion mutant constructs were infiltrated into *N. benthamiana* leaves, which were then inoculated by *P. infestans* strain 88069 1 d after *A. tumefaciens* infiltration. Lesion sizes were determined and photographed at 7 d post‐infection.

### Confocal fluorescence microscopy

*Nicotiana benthamiana* leaf cells of wild‐type (WT), GFP‐LTi6b or mOrange‐LTi6b PM marker‐expressing transgenic lines (Kurup *et al*., 2005; Yang *et al*., 2016) were infiltrated with *A. tumefaciens* containing expression constructs at OD_600_ of 0.01. Cells transiently expressing SFI5 fusion constructs were imaged 48 h post‐infiltration on Zeiss 710 or Nikon A1R confocal microscopes. GFP was excited with 488 nm light and the emissions were detected between 500 and 530 nm. mRFP and mOrange were excited with 561 nm light and emissions were detected between 600 and 630 nm or 580 and 610 nm, respectively. Cells displaying a low intensity of GFP fluorescence were imaged to minimize artefacts of overexpression. For co‐expression of the GFP‐SFI5 constructs with the cytoplasmic mRFP control construct, *A. tumefaciens* suspensions were pre‐mixed before infiltration.

Images were processed with propriety confocal software or ImageJ as required. Figures were constructed with Adobe Photoshop and Adobe Illustrator.

## Results

### SFI5 binds CaM *in vitro* in the presence of Ca^2+^

**Figure 1:**
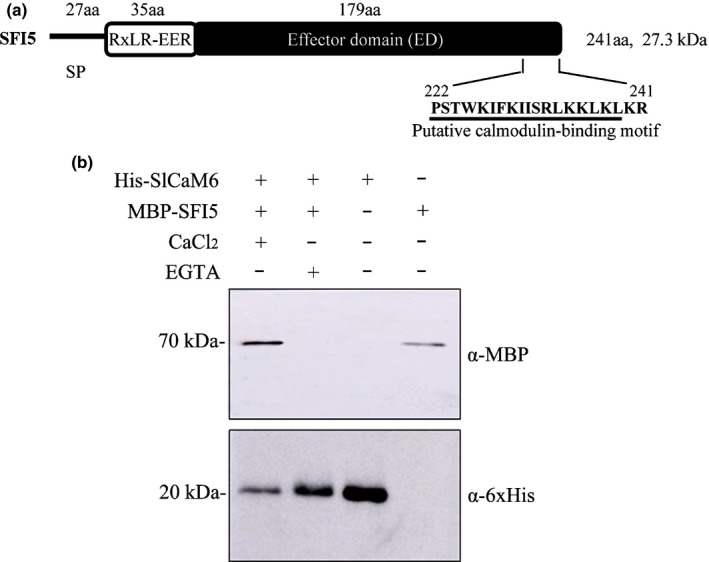
SFI5 binds calmodulin (CaM) in the presence of Ca^2+^. (a) Schematic representation of the SFI5 effector, showing the predicted amino acid (aa) length of the signal peptide (SP), host‐translocation motif‐containing domain (RxLR‐EER) and effector domain (ED). The putative CaM‐binding motif is underlined. The CaM‐binding motif was predicted in the Calmodulin Target Database (Yap *et al*., 2000). (b) Pull‐down assay of SFI5 and SlCaM6. MBP‐tagged SFI5 (MBP‐SFI5) and 6xHis‐tagged SlCaM6 (His‐SlCaM6) were mixed with 5 mM CaCl_2_ or 5 mM EGTA in PBS buffer and incubated for 1 h at 4°C. The mixtures were then incubated with Ni‐NTA agarose beads for 30 min, followed by extensive wash with wash buffer of Ni‐NTA beads. The bound proteins were eluted with 1 M imidazole and mixed with SDS sample buffer for SDS‐PAGE. Gel‐separated proteins were transferred to nitrocellulose membranes and immunodetected with anti‐MBP and anti‐6xHis antibodies, respectively. Purified MBP‐SFI5 and His‐SlCaM6 were used as controls.

*Phytophthora infestans* SFI5 is a 241‐amino acid protein bearing the typical signature of RXLR effectors with an N‐terminal signal peptide, followed by a sequence (Ala^28^ to Arg^62^) containing the RXLR motif and a predicted C‐terminal effector domain of 178 amino acid residues (Phe^63^ to Arg^241^). Sequence analysis in the Calmodulin Target Database (Yap *et al*., 2000) revealed a putative CaM‐binding site located at the C‐terminal end of SFI5 including 18 amino acids between Pro^222^ and Leu^239^ (Fig. 1a). A genome‐wide analysis has revealed six *CaM* genes in tomato, which encode four isoforms – SlCaM1, SlCaM2, SlCaM3/4/5 and SlCaM6 – sharing 91–99% sequence identity (Zhao *et al*., 2013) (Fig. S1). We cloned *SlCaM1*,* SlCaM3/4/5* and *SlCaM6* but not *SlCaM2* from a tomato cDNA library.

We performed a pull‐down assay to analyse *in vitro* the interaction between SFI5 and CaM. The purified His‐SlCaM6 and MBP‐SFI5 proteins were expressed in *E. coli* and purified prior to interaction studies using Ni‐NTA affinity chromatography in the presence of Ca^2+^ or the Ca^2+^ chelator EGTA. As shown in Fig. 1(b), in the presence of Ca^2+^, both a 70 kDa band corresponding to MBP‐SFI5 and a 20 kDa band corresponding to His‐SlCaM6 were immunodetected in the Ni‐NTA eluate (Lane 1 of Fig. 1b). In the presence of EGTA, the 70 kDa MBP‐SFI5 was not detected (Lane 2 of Fig. 1b). Immunodetection of the purified His‐SlCaM6 and MBP‐SFI5 controls in lanes 3 and 4 of Fig. 1(b), confirmed the identity of the bands in lanes 1 and 2. Thus, we conclude that SFI5 interacts with SlCaM6 *in vitro* in a Ca^2+^‐dependent manner.

### A C‐terminal 17‐aa peptide of SFI5 binds CaM *in vitro* in the presence of Ca^2+^

**Figure 2:**
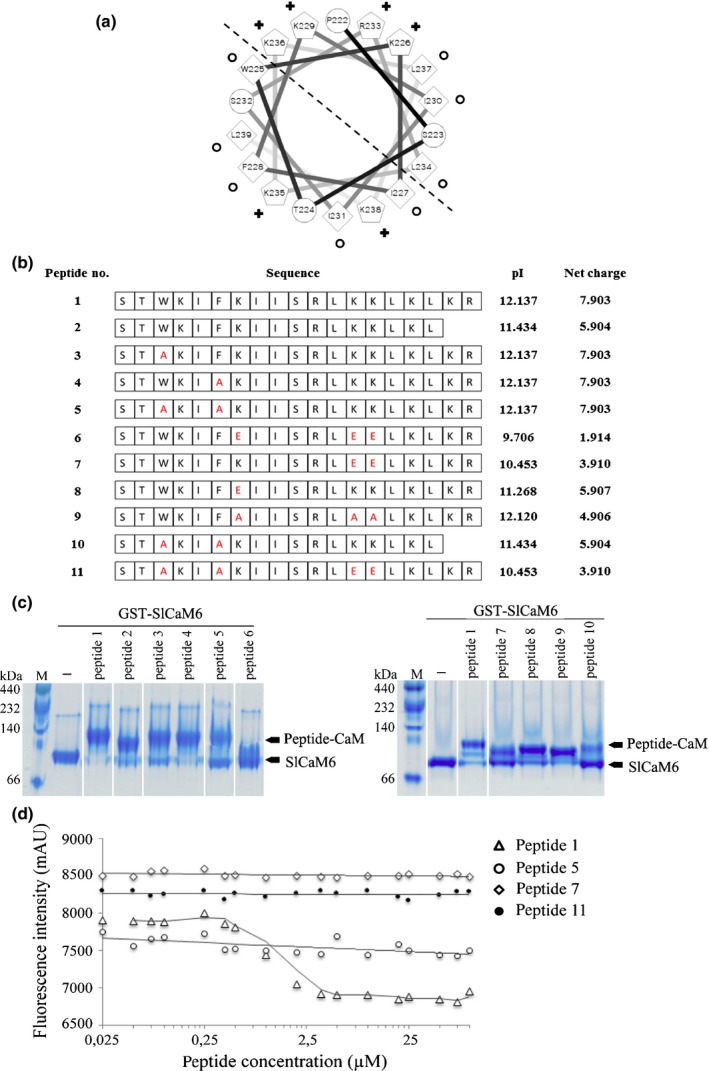
*In vitro* interaction between C‐terminal 17‐aa domain of SFI5 and GST‐SlCaM6. (a) Helical wheel projection of the predicted 18‐amino acid calmodulin (CaM)‐binding domain of SFI5 (Pro^222^ to Leu^239^). Hydrophobic and potentially positively charged residues are marked with circles and crosses, respectively. The dashed line divides the amphipathic helix into the hydrophobic side and hydrophilic side. Numbers refer to amino acid positions in SFI5 protein. (b) Synthetic peptides corresponding to the predicted CaM‐binding site with various amino acid mutations. Peptide 1 represents the last 19 aa at the C‐terminus of SFI5. Peptides 2–11 are truncated or mutated versions of peptide 1, in which the substituted amino acids are presented in red. The pI and net charge (at pH7.0) of each peptide were calculated by Editseq (Lasergene v.8; dnastar). (c) CaM mobility shift on tris/glycine native gels. Purified GST‐SlCaM6 (50 μM) was incubated with each synthetic peptide (133 μM) in the presence of 5 mM CaCl_2_. Samples were separated on tris/glycine native gels followed by Coomasssie blue staining. Arrows indicate the position of bands representing free GST‐SlCaM6 (SlCaM6) and GST‐SlCaM6‐peptide complex (Peptide‐CaM). (d) 1‐Anilinonaphthalene‐8‐sulfonate (ANS) fluorescence competition assay. GST‐SlCaM6 (1 μM), ANS (100 μM) in the buffer containing 20 mM Tris‐HCl pH7.5, 100 mM NaCl, 1 mM CaCl_2_ were incubated with increasing concentration of selected peptides for 30 min, and kinetic changes of fluorescence was monitored at an excitation wavelength of 360 nm (λ_ex_) and an emission wavelength of 460 nm (λ_em_). Data points represent the mean ± SE of three technical replicates from three independent assays.

The predicted CaM‐binding site at the C‐terminus of SFI5 does not contain a canonical CaM‐binding motif (Mruk *et al*., 2014). However, a helical wheel projection of the 18‐amino acid stretch (Pro^222^ to Leu^239^) showed a basic amphipathic structure with one side enriched in positively charged residues (Lys^226^, Lys^229^, Arg^233^, Lys^236^) and the opposite side rich in hydrophobic residues (Trp^225^, Ile^227^, Phe^228^, Ile^231^), which resembles typical CaM recognition and binding motifs (Fig. 2a). Peptides corresponding to the predicted CaM‐binding site of SFI5 and with amino acid mutations, which led to different net charges, were synthesized for CaM‐binding analysis (Figs 2b, S2).

Complex formation between GST‐SlCaM6 and synthetic peptides was shown by gel shift experiments on tris‐glycine native gels. All peptides were mixed with recombinant GST‐SlCaM6 in the presence of Ca^2+^. As shown in Fig. 2(c), mixing peptide 1 with GST‐SlCaM6 resulted in a band shift to a higher molecular weight compared to the GST‐SlCaM6 control, indicating complex formation between the predicted CaM‐binding motif and SlCaM6. Peptide 2, in which the last two amino acid residues (Lys^240^‐Arg^241^) of peptide 1 were removed, was able to bind SlCaM6 with nearly the same efficacy as peptide 1, although its net charge at pH 7 decreased from + 7.9 to + 5.9 (Fig. 2b). This suggests that these two residues (Lys^240^‐Arg^241^) are not crucial for CaM association. Replacing two hydrophobic residues, Trp^225^ and Phe^228^, with Ala in peptides 3 and 4, respectively, did not severely affect the complex formation with SlCaM6. However, replacing both Trp^225^ and Phe^228^ with Ala significantly reduced the binding affinity of peptide 5 and peptide 10 to SlCaM6, with *c*. 50–75% of the SlCaM6 remaining in an unbound state. The three lysine residues Lys^229^, Lys^235^ and Lys^236^ were predicted by computer modelling to be important for direct interaction with CaM (Yap *et al*., 2000; Mruk *et al*., 2014). Replacement of these lysine residues with the negatively charged residue Glu caused a drastic reduction of the net charge of peptide 6 and abolished the interaction with SlCaM6. The replacement of Lys^235^ and Lys^236^ only with Glu (peptide 7) or Lys^229^ only with Glu (peptide 8) had less effect on the net charge and resulted in an increase of peptide binding to SlCaM6 (Fig. 2b,c). Interestingly, replacing the three lysine residues with alanine in peptide 9 also caused less reduction of the net charge and did not affect peptide binding to SlCaM6.

The gel shift assay also was performed with His‐tagged SlCaM6 and additional synthetic peptides with mutations were tested (Fig. S2). The replacement of both Trp^225^ and Phe^228^ with His significantly reduced, but did not abolish the binding of peptide 12 to SlCaM6. No binding was detected when Trp^225^ and Phe^228^ were replaced with Glu (peptide 13), or in the quadruple mutant, in which Trp^225^, Phe^228^, Lys^235^ and Lys^236^ were replaced by Glu (peptide 14). The results with His‐SlCaM6 are highly consistent with those of the assay with GST‐tagged SlCaM6. In summary, the net charge of the C‐terminal helix of SFI5 is a crucial factor for CaM‐binding and Trp^225^, Phe^228,^ Lys^235^ and Lys^236^ are important residues for the interaction.

To underpin the importance of the two hydrophobic residues (Trp^225^ and Phe^228^) and the two basic residues (Lys^235^ and Lys^236^) for CaM‐binding, we carried out CaM competition assays with four synthetic peptides (1, 5, 7 and 11) and 1‐Anilinonaphthalene‐8‐sulfonate (ANS), a compound with high affinity to CaM which is fluorescent upon binding. The kinetics of ANS fluorescence change was monitored in the presence of increasing concentrations of different peptides. As shown in Fig. 2(d), the fluorescence curve rapidly declined with increasing concentration of peptide 1 (IC_50_ = 1 μM), indicating that competition occurred between ANS and the peptide 1 for SlCaM6‐binding. By contrast, ANS fluorescence decreased smoothly or remained unchanged with increasing concentrations of peptide 5, 7 and 11 (the last a quadruple mutant of Trp^225^, Phe^228^, Lys^235^ and Lys^236^). This result confirmed the importance of the mutated amino acids within the CaM‐binding site of SFI5.

### The CaM binding motif of SFI5 is necessary for the interaction with tomato CaMs *in vivo*

**Figure 3:**
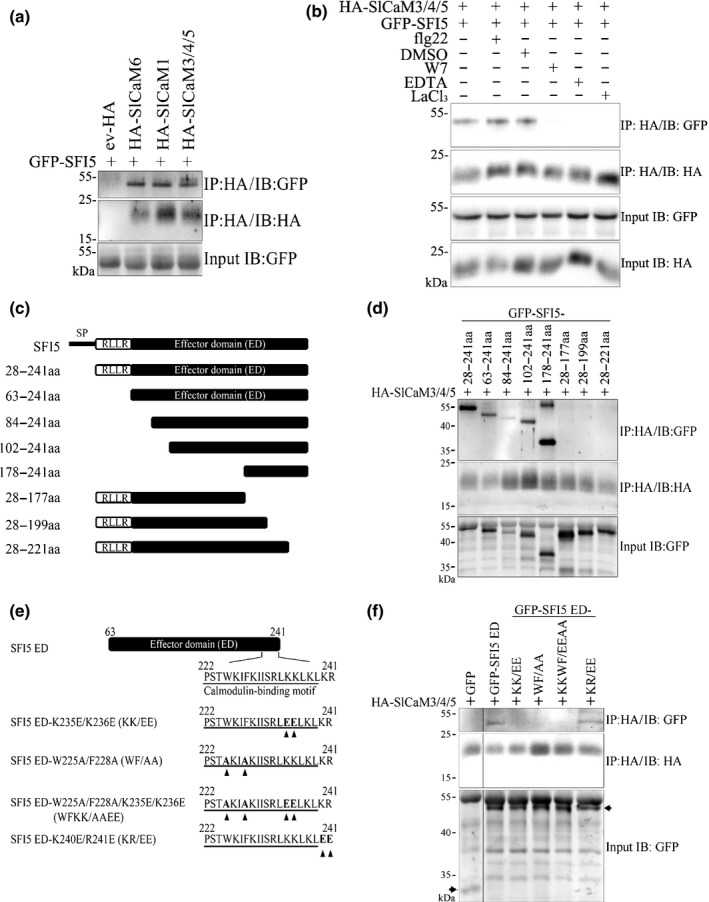
SFI5 interacts with tomato calmodulins (CaMs) *in vivo*. (a) Co‐immunoprecipitation analysis of transiently expressed GFP‐SFI5 and HA‐SlCaMs in tomato protoplasts. Extracted proteins (input) were subjected to immunoprecipitation (IP) with anti‐HA affinity matrix followed by immunobloting (IB) with anti‐HA antibody to detect the tomato CaMs and anti‐GFP antibody to detect the SFI5 fusion. (b) Co‐immunoprecipitation analysis of transiently expressed GFP‐SFI5 and HA‐SlCaM3/4/5 in tomato protoplasts upon different treatments. Protoplast samples were treated with 500 nM flg22, 0.75% DMSO (mock), 250 nM W7 (stock solution: 33 mM W7 dissolved in 99.5% DMSO) or 1 mM LaCl_3_ before the protoplasts were harvested for the IP with anti‐HA antibody. For EDTA treatment, total proteins were extracted with IP buffer containing 20 mM EDTA. (c) Schematic diagrams of SFI5 deletion mutants. Numbers indicate positions of amino acid (aa) residues based on the full‐length protein sequence. (d) Co‐immunoprecipitation analysis of transiently expressed GFP‐SFI5 deletion mutants and HA‐SlCaM3/4/5 in tomato protoplasts. (e) Schematic diagrams of site‐directed mutants of SFI5 effector domain (ED). Numbers indicate the amino acid positions based on the full‐length protein sequence. KK/EE, WF/AA, WFKK/AAEE and KR/EE correspond to amino acid exchanges of Lys^235^ and Lys^236^ with Glu, Trp^225^ and Phe^228^ with Ala, Lys^235^ and Lys^236^ with Glu and Trp^225^ and Phe^228^ with Ala, and Lys^240^ and Arg^241^ with Glu, respectively. Mutations are indicated by arrowheads (f) Co‐immunoprecipitation analysis of transiently expressed GFP‐SFI5 ED site‐directed mutants with HA‐SlCaM3/4/5 in tomato protoplasts. Bands corresponding to GFP and GFP‐tagged SFI5 ED point mutants are indicated by arrows. These results are representative of three replicates. GFP, green fluorescent protein.

In order to find out the host targets of SFI5 and confirm the results of the *in vitro* interaction between SFI5 and SlCaM6, we transiently expressed N‐terminal HA‐tagged SFI5 in tomato protoplasts. Total proteins of transgenic tomato protoplasts were isolated and immunoprecipitated by anti‐HA beads followed by LC‐MS/MS analysis. Through this approach, we identified a number of peptides that match tomato CaMs among the proteins isolated from protoplasts expressing HA‐SFI5 (Table S2). CaMs were not identified in immunoprecipitated material from protoplasts expressing HA‐SFI1, another MTI‐suppressing RXLR effector of *P. infestans* (Zheng *et al*., 2014) or transformed with the empty vector control (Table S2). In the next step, we performed pairwise interaction studies with SFI5 and SlCaM1, SlCaM3/4/5 and SlCaM6 in tomato protoplasts. GFP‐tagged SFI5 (GFP‐SFI5) and HA‐tagged CaMs were co‐expressed in tomato protoplasts. Total protein extracts from transformed protoplasts were prepared and immunoprecipitated with anti‐HA antibody‐coupled beads, followed by immunoblotting with anti‐GFP and anti‐HA antibodies, respectively. The result in Fig. 3(a) shows that GFP‐tagged SFI5 associated with all HA‐tagged CaMs without apparent specificity. To confirm whether the interaction between SFI5 and CaMs relies on the presence of Ca^2+^ and MAMP perception, we performed co‐immunoprecipitation assays with tomato protoplasts expressing HA‐SlCaM3/4/5 and GFP‐SFI5 treated with flg22, a CaM antagonist N‐(6‐aminohexyl)‐5‐chloro‐1‐naphthalene‐sulphonamide (W7) or Ca^2+^ channel blockers (LaCl_3_ and EDTA). The results in Fig. 3(b) show that the protein association was independent of the flg22 treatment but significantly inhibited by the presence of CaM antagonist and Ca^2+^ channel blockers.

We used the results gained from the *in vitro* binding assay to define the molecular determinants of the interaction between GFP‐SFI5 and SlCaM3/4/5 in tomato protoplasts. We generated a series of N‐terminal or C‐terminal deletion constructs of SFI5 and employed site‐directed mutagenesis to replace the four key residues (Trp^225^, Phe^228^, Lys^235^ and Lys^236^) in order to validate their role in the interaction between SFI5 and SlCaMs *in vivo* (Fig. 3c,e). After co‐immunoprecipitation and Western blot analysis, SlCaM3/4/5 interactions were detected with all N‐terminal deletion mutants of SFI5 including the shortest truncated protein corresponding to the last 63 amino acids (178–241 aa). By contrast, none of the three C‐terminal deletion variants, including the one lacking only the amphipathic helix of 19 amino acids (28–221 aa), was able to associate with HA‐SlCaM3/4/5 (Fig. 3d). The SFI5 variants carrying mutations in two hydrophobic residues (Trp^225^Ala/Phe^228^Ala – WF/AA), basic residues (Lys^235^Glu/Lys^236^Glu – KK/EE) and quadruple residues (Trp^225^Ala/Phe^228^Ala/Lys^235^Glu/Lys^236^Glu – WFKK/AAEE) failed to associate with HA‐SlCaM3 (Fig. 3e,f). As anticipated, the variant in which the last two amino acids (Lys^240^ and Arg^241^) were replaced by glutamic acid (KR/EE) did not affect CaM‐binding (Fig. 3e,f).

From the *in vitro* and *in vivo* interaction assays, we concluded that SFI5 has a unique CaM‐binding site formed by a 17‐aa core region (Ser^223^ to Leu^239^) containing an alpha helical fold and amphipathic properties, in which the two hydrophobic residues (Trp^225^ and Phe^228^) and the two basic residues (Lys^235^ and Lys^236^) play critical roles in binding CaMs.

### The CaM‐binding motif participates in the plasma membrane localization of SFI5

**Figure 4:**
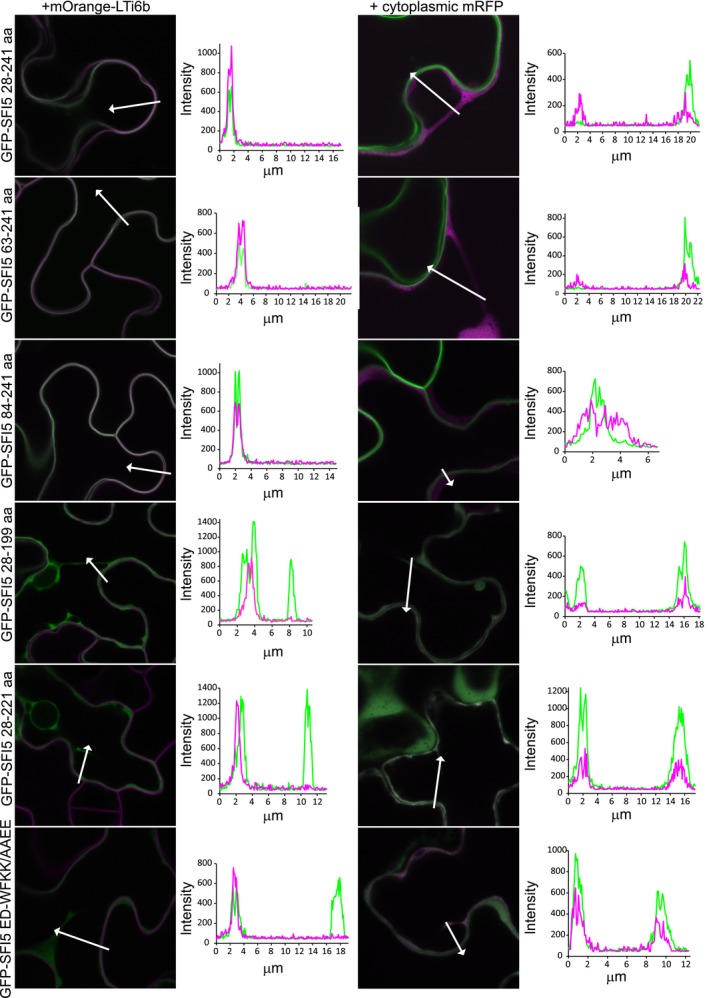
The calmodulin (CaM) binding motif of SFI5 is required for plasma membrane localization in *Nicotiana benthamiana* leaves. Single optical section images of GFP‐tagged SFI5 deletion and mutant forms (as indicated to the left of the images) coexpressed with the PM marker mOrange‐LTi6b or cytoplasmic mRFP (as indicated above the image columns). The fluorophores are shown in green for GFP and magenta for the mRFP or mOrange. The arrows indicate the lines drawn to generate the intensity profiles shown to the right of each image. Each profile was drawn such that both a cytoplasmic strand, or region of cytoplasm, and the plasma membrane were crossed. The lengths of the profiles are shown in the graphs in μm and the intensities are as generated by the confocal software. GFP, green fluorescent protein.

GFP‐SFI5 is located mainly at the plasma membrane (PM) in tomato protoplasts and *N. benthamiana* leaves, with very little fluorescence detectable in the cytoplasm (Zheng *et al*., 2014). To determine whether the CaM‐binding site is important for the intracellular localization of SFI5, full length SFI5 and N‐terminal or C‐terminal deletion variants or amino acid point mutants (Fig. 3c,e) were coexpressed with a PM marker, mOrange‐LTi6b, or cytoplasmic mRFP. Laser scanning confocal microscopy imaging shows that all three GFP‐tagged N‐terminal deletion forms of SFI5 used in this analysis (GFP‐SFI5 28–241 aa, GFP‐SFI5 63–241 aa and GFP‐SFI5 84–241 aa) were predominantly localized at the PM, as illustrated by the overlap of the GFP and mOrange fluorescence signals (Fig. 4), and the failure of the GFP fluorescence to overlap with free mRFP signal. This indicates that the N‐terminal region of SFI5 is not required for the plasma membrane localization. By contrast, the two GFP‐tagged C‐terminal deletion variants (GFP‐SFI5 28–199 aa and GFP‐SFI5 28–221 aa) and the quadruple point mutant (GFP‐SFI5 ED‐WFKK/AAEE) of SFI5 localized predominantly in the cytosol, with GFP fluorescent peaks representing trans‐vacuolar cytoplasmic strands, and a minimal overlap with mOrange‐LTi6b (Fig. 4). This suggests that the PM‐association of SFI5 is dependent on the C‐terminal CaM‐binding motif. Mutation of the last two amino acids (KR/EE) increased the cytoplasmic signal from the GFP fusion (GFP‐SFI5 ED KR/EE) but did not abolish the PM localization (Fig. S3). Co‐localization studies performed in tomato protoplasts using the aforementioned deletion and point mutants of SFI5 and the bacterial effector AvrPto, which is associated with the PM through the presence of an N‐terminal myristoylation site (Shan *et al*., 2000; He *et al*., 2006), mirrored the results obtained in *N. benthamiana* leaves (Fig. S4). Next, we investigated whether SFI5 and CaM interact at the PM. Co‐expression of mRFP‐SlCaM3/4/5 and GFP‐SFI5 28‐241 aa showed an absence of signal overlap, suggesting that if CaM binding is required for the PM localisation of SFI5, the interaction does not occur at the PM and that CaM likely dissociates from SFI5 before it locates at the PM (Fig. S5).

### Both the C‐terminal CaM‐binding motif and N‐terminal region of SFI5 are required for effector activity

SFI5 is an effector that disturbs the earliest signalling events of MTI responses, including flg22‐dependent *pFRK1‐Luc* reporter gene expression, post‐translational activation of SlMPK1 and SlMPK3, and ROS production, and thereby promotes *P. infestans* infection in plant leaves (Zheng *et al*., 2014). To investigate the importance of the CaM‐binding site of SFI5 for disruption of MTI responses, HA‐tagged SFI5 deletion and point mutation variants were transiently expressed in tomato protoplasts (Fig. S6) and tested in a set of bio‐assays to measure early immune responses triggered by flg22.

**Figure 5:**
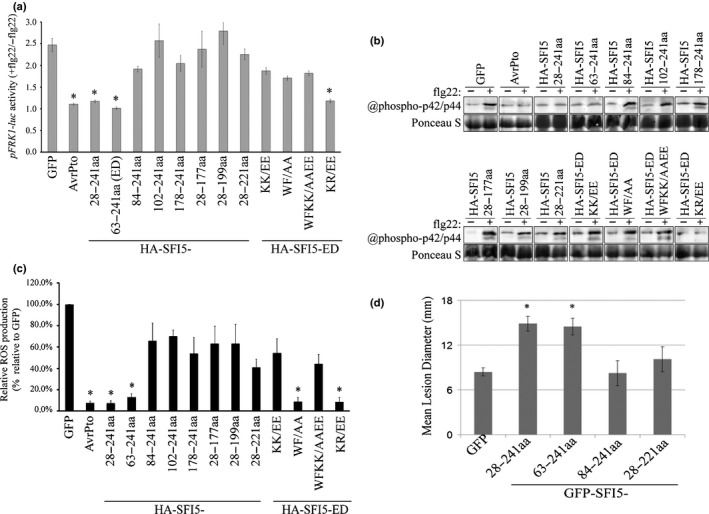
Both the calmodulin (CaM) binding motif and N‐terminal region of SFI5 are required for the suppression of flg22‐triggered early immune responses in tomato protoplasts and *Phytophthora infestans* infection in *Nicotiana benthamiana*. (a) Tomato protoplasts coexpressing HA‐tagged SFI5 deletion or point mutants with the two reporter genes *pFRK1‐Luc* and *pUBQ10‐GUS* were treated with or without flg22 (+/−flg22) and the *pFRK1‐Luc* activity was measured after 3 h. The promoter activity is calculated as the ratio of flg22‐induced luciferase activity relative to the untreated sample, which was normalized to the internal GUS activities (*pFRK1‐Luc* activity +flg22/−flg22). GFP and AvrPto served respectively as a negative and positive control for the suppression of *pFRK1‐Luc* activation by flg22. (b) Tomato protoplasts expressing HA‐tagged SFI5 deletion or point mutants were collected 20 min after flg22 treatment (+) or without flg22 treatment (−), and the phosphorylated mitogen‐activated protein kinases MAPK were detected by immunoblotting with the antibody raised against phosphorylated MAPK p44/p42. Ponceau S staining is shown as a loading control. (c) The oxidative burst in tomato protoplasts expressing HA‐tagged SFI5 deletion or point mutants is represented as the percentage of total photon counts measured between 6 and 20 min after flg22 treatment of the GFP control, which was set to 100%. GFP and AvrPto served respectively as negative and positive controls for the suppression of *pFRK1‐Luc* and MAPK activation and ROS burst by flg22. Data in (a, c) represent the mean ± SE from four independent experiments, for each of which three technical replicates were carried out. *, *P *<* *0.05 (one‐way ANOVA followed by Dunnett's multiple comparison test). The results in (b) are representative of at least three independent experiments. (d) N‐terminal or C‐terminal deletion mutants of SFI5 were each transiently expressed via agro‐infiltration in one half of a *N. benthamiana* leaves and empty vector (GFP) was expressed in the other half. After 24 h, the infiltrated leaves were inoculated with *P. infestans*. Disease lesion diameters were measured 7 d post‐inoculation. Results are mean ± SE from three biological replicates, each of which used 24 leaves for inoculation per construct. Significant difference (*, *P *<* *0.01) in lesion size compared to empty vector control was determined by one‐way ANOVA. GFP, green fluorescent protein.

First, we measured the impact of truncated and mutated SFI5 proteins on the induction of the *pFRK1‐Luc* reporter gene expression after flg22 treatment using the method described in Zheng *et al*. (2014). As shown in Fig. 5(a), expressing SFI5 C‐terminal deletion variants (HA‐SFI5 28‐177 aa, HA‐SFI5 28‐199 aa or HA‐SFI5 28–221 aa) failed to block flg22‐induced *Luc* activity in comparison to expressing SFI5 (HA‐SFI5 28–241 aa) or AvrPto. Point mutations in the CaM‐binding region (HA‐SFI5 ED‐KK/EE, HA‐SFI5 ED‐WF/AA or HA‐SFI5 ED‐WFKK/AAEE) also reduced the ability of SFI5 to block flg22‐triggered reporter gene activation, although the reduction was less than that of the deletion mutants. The HA‐SFI5 ED‐KR/EE mutant was as active as the positive controls (Fig. 5a). Interestingly, suppression of the flg22‐mediated reporter gene activation also was attenuated in tomato protoplasts expressing N‐terminal deletion constructs of SFI5 (HA‐SFI5 84–241 aa, HA‐SFI5 102–241 aa or HA‐SFI5 178–241 aa), except for the one lacking only the RXLR motif (HA‐SFI5 63‐241aa) which retained full suppression activity (Fig. 5a).

Second, immunodetection of flg22‐dependent SlMPK1 and SlMPK3 activation using the p44/p42 antibody was performed with tomato protoplasts expressing the SFI5 deletion and point‐mutation constructs. The results shown in Fig. 5(b) are consistent with the *pFRK1‐Luc* induction assay.

Third, the results of the oxidative burst assays in tomato protoplasts expressing SFI5 deletion and point mutation constructs were largely in agreement with the results of *pFRK1‐Luc* induction and MAPK activation assays. Notably, the HA‐SFI5 mutant carrying a substitution for the two hydrophobic residues (HA‐SFI5 ED‐WF/AA) retained the ability to repress ROS production under the defined experimental conditions (Fig. 5c).

In order to further determine the role of the N‐terminal and CaM binding domains in the virulence function of SFI5, two GFP‐SFI5 variants with an N‐terminal deletion (63–241 aa and 84–241 aa) and one with a C‐terminal deletion (28–221 aa) were transiently expressed in *N. benthamiana* leaves followed by inoculation of *P. infestans*. As anticipated, both SFI5 (28–241 aa)‐expressing leaves and effector domain (63–241 aa)‐expressing leaves showed significantly larger lesion size compared to those on the GFP expressing control leaves at 7 d post‐pathogen inoculation. By contrast, leaves expressing two SFI5 deletion variants (84–241 aa and 28–221 aa) did not enhance the susceptibility to *P. infestans* infection since the lesion sizes were similar to those on control leaves (Fig. 5d). Altogether, these results (summarized in Table 1) indicate that there is a correlation between CaM binding, suppression of early MTI signalling and virulence function of SFI5. In addition, another domain of unknown function, located at the N‐terminal part of SFI5, after the RXLR motif and spanning *c*. 20 aa residues (Phe^63^‐Lys^84^), is required for the activity and function of SFI5 in the host cell.

**Table 1:** Overview of *in vivo* Ca^2+^/calmodulin (CaM)‐binding, localization, and microbe‐associated molecular pattern (MAMP)‐triggered immunity (MTI)‐suppression of truncated or mutated SFI5

SFI5	Domain	CaM‐binding	PM‐localization	FRK1‐suppression	MAPK suppression	ROS suppression
	28–241	+	+	+	+	+
	63–241	+	+	+	+	+
	84–241	+	+	−	−	−
	102–241	+	Not tested	−	−	−
	178–241	+	Not tested	−	−	−
	28–177	−	Not tested	−	−	−
	28–199	−	−	−	−	−
	28–221	−	−	−	−	−
SFI5 ED	Mutation	CaM‐binding	PM‐localization	FRK1‐suppression	MAPK suppression	ROS suppression
	WF/AA	±	Not tested	−	−	+
	KK/EE	±	Not tested	−	−	−
	WFKK/AAEE	−	−	−	−	−
	KR/EE	+	+	+	+	+

MAPK, mitogen‐activated protein kinase; PM, plasma membrane; ROS, reactive oxygen species.

## Discussion

*Phytophthora infestans* is an oomycete pathogen that infects the crop plants tomato and potato in nature, and the model plant *Nicotiana benthamiana* in the laboratory (Haas *et al*., 2009; King *et al*., 2014; Whisson *et al*., 2016). To colonize host plants, *P. infestans* secretes effector proteins, many of which contain an RxLR motif downstream of the N‐terminal signal peptide for translocation into the plant cell and a C‐terminal effector domain several of which have been shown to suppress plant immunity. So far, there are no studies showing that RXLR effectors suppress immunity by interacting with Ca^2+^ signalling components. Thus, SFI5 is the first example of an oomycete effector that suppresses microbe‐associated molecular patterns (MAMP)‐triggered immunity (MTI) responses by interacting with calmodulin (CaM).

The structure–function analysis presented here has accurately delimited the domain and residues of SFI5 that are involved in CaM binding. The C‐terminal 18 aa‐region of SFI5 forms an amphipathic α‐helix wheel with the segregation of basic and hydrophobic residues on opposite sides (Fig. 2a), characteristic of CaM‐binding domains (O'Neil & DeGrado, 1990; Meador *et al*., 1992; Crivici & Ikura, 1995). The CaM‐binding site of SFI5 appears to be noncanonical due to the weak similarity to any known CaM‐binding domains. To verify the CaM‐binding ability, peptides corresponding to the 18 aa‐region were tested for binding with SlCaM6 from tomato by tris‐glycine native gels (Fig. 2b,c). The results showed that the predicted CaM‐binding site of SFI5 is functional, which was confirmed further by *in vitro* and *in vivo* interaction assays with full‐length SFI5 and CaMs from tomato (Fig. 3a). The two hydrophobic residues (Trp^225^ and Phe^228^) are critical anchor residues but other hydrophobic residues at the same side of the α‐helix wheel (Ile^227^, Ile^231^ or Leu^239^) might also be important for the interaction with CaM. It is worth noting that mutation of the Trp^225^ and Phe^228^ residues did not dramatically affect the suppression of flg22‐triggered oxidative burst (Fig. 5c). The 1‐Anilinonaphthalene‐8‐sulfonate (ANS) fluorescence competition assay with peptide 5 (WF/AA) suggests abrogation of the interaction with CaM (Fig. 2d) but the gel shift assay showed a residual complex formation (Fig. 2c). It is therefore possible that replacement of the two strong hydrophobic amino acids with a weak hydrophobic residue (alanine) compromised, but did not completely abolish CaM‐binding activity of SFI5 *in vivo*, which was sufficient to suppress the oxidative burst. Additional Trp^225^/Phe^228^ mutant peptides, in which these two hydrophobic residues were converted into histidine or glutamate residues, did not bind to CaM (Fig. S2a,b). Dose–response assays with those SFI5 variants would help to better quantify CaM binding and their impact on the biological response. This would confirm the importance of CaM binding for the MTI‐suppressing activity of SFI5.

The molecular determinants of CaM that are required for the interaction with SFI5 are unknown. CaMs and CaMs‐like (CMLs) form a highly conserved Ca^2+^ sensor protein family, which is present in all eukaryotes. The 3D structure of CaM has the hallmark of a dumbbell shape with four EF‐hand Ca^2+^‐binding motifs organized in pairs and embedded in two globular domains separated by a long flexible helix. After Ca^2+^ binding, CaM undergoes conformational changes that expose its hydrophobic surfaces and subsequently interacts with a large array of proteins that are implicated in many cellular processes (Bouche *et al*., 2005; McCormack *et al*., 2005). It would be interesting to solve the crystal structure of SFI5 in complex with CaM, which could provide new insights on the structural variability of CaM/CaM‐binding protein interactions and on the mode of action of SFI5 *in planta*.

The CaM‐binding motif of SFI5 is required for the plasma membrane localization, MTI‐suppression and for promoting *P. infestans* colonization (Fig. 5; Table 1) but we did not observe CaM‐SFI5 co‐localization at the plasma membrane (Fig. S5). A simple and likely explanation for this is that CaM binding and activation may be required for SFI5 to interact subsequently with operative targets that are localized at the plasma membrane (PM). However, dissociation of CaM from SFI5 may be required for it to interact with the operative targets. *In vivo* Förster resonance energy transfer‐fluorescence lifetime imaging microscopy (FRET‐FLIM) would provide an alternative approach to study the interaction between SFI5 and CaM and perhaps provide information on the dynamics of their coupling. This technique has been successfully applied to study the spatiotemporal interaction dynamics of barley Mildew resistance Locus O (MLO) with its activator, CaM. It revealed that an increasing number of MLO/CaM complexes in the vicinity of the penetration sites was coincident with successful pathogen entry into host cells (Bhat *et al*., 2005).

Bioinformatic analyses do not predict the presence of a transmembrane or membrane‐anchoring domain in the sequence of SFI5. To further determine the importance and the role of CaM‐mediated PM‐localization for the SFI5 MTI‐suppressing activity, a PM anchor myristoylation site could be introduced into SFI5 and SFI5 variants deficient in CaM‐binding and the resulting proteins could be tested for their MTI‐suppressing activity. We have used this targeting approach to mis‐locate the RXLR effector SFI1 from the nucleus to the plasma membrane to thereby demonstrate that SFI1 must enter the nucleus to suppress MTI (Zheng *et al*., 2014). However, it has not been used to investigate whether simple restoration of PM localization would restore function.

Although we provide strong correlative evidence that CaM‐binding is required for full function of SFI5, it is still unclear how SFI5 interferes with MTI signalling. One possibility is that SFI5 directly targets and inhibits the function of CaM or CMLs that regulate the activity of downstream CaM‐ and CML‐binding proteins which have a positive role on MTI. Large scale protein–protein interaction screens combined with the availability of genome and transcriptome resources have revealed a considerable number of putative CaM/CML‐binding proteins implicated in the regulation of plant immunity. Experimental evidence includes the finding that one member of the CaM‐binding transcription activator (CAMTA) family in Arabidopsis, AtCAMTA3 (also designated as AtSR1), was shown to suppress the expression of genes of the salicylic acid (SA) biosynthetic pathway thereby repressing SA‐dependent plant defence against bacteria and fungi (Galon *et al*., 2008; Du *et al*., 2009). Barley MLO, a PM‐resident protein, requires Ca^2+^/CaM association to compromise plant defence against powdery mildew (Kim *et al*., 2002). In addition, AtCaM7 was recently shown to interact and to co‐localize with ATP‐binding cassette (ABC) transporter PENETRATION 3 (PEN3) at the plasma membrane‐cytoplasm interface and may be involved in PEN3‐mediated nonhost resistance (Campe *et al*., 2016), whereas PEN3 focal accumulation was elicited upon MAMP perception or infection by adapted bacterial pathogens (Underwood & Somerville, 2013; Xin & He, 2013). However, the observation that functional SFI5 does not co‐localize with CaM at the PM potentially favours additional unknown proteins at the PM being targeted to suppress MTI.

There are few publications reporting an interaction of CaMs/CMLs with microbial effectors. Recently, HopE1 from *P. syringae* was discovered to interact with CaM and this interaction was required for further association with host microtubule‐associated protein 65 (MAP65). Upon association, MAP65 dissociates from the microtubule network which is thought to cause suppression of MAMP‐induced pathogenesis‐related protein secretion and enhanced susceptibility to bacterial infection (Guo *et al*., 2016). To date, it is unclear how HopE1 manipulates MAP65, but a conclusion of the authors was that CaM serves as a factor to activate HopE1 function in host cells. This is similar to our hypothesis that CaM is not the operative target of SFI5 but that SFI5 utilizes plant CaMs as positive regulators of its effector activity after translocation into the host cell. Given that SFI5 does not display specificity in binding distinct CaMs from tomato and Arabidopsis, it is possible that SFI5 association with CaM promotes the interaction with other CaM‐binding components. In this respect, the identification of potential additional targets might provide new findings on the mode of action SFI5.

The finding of a physical association of SFI5 with different CaM isoforms is the first direct link between an oomycete plant pathogen effector and components of Ca^2+^/CaM signalling in plants. Our current model predicts that SFI5 activation in host cells requires the association with CaM, in a Ca^2+^‐dependent manner, at the C‐terminal Pro^222^‐Leu^239^ alpha helix, triggering a conformational change of SFI5 conferring its ability to affect MAMP signal transduction pathways by manipulating one or several unknown membrane‐associated proteins, likely pattern recognition receptors and/or signalling components. Further molecular and biochemical studies are needed to dissect the specific mode of action of SFI5 in host cells and to unravel the molecular basis of the nonfunctionality of SFI5 in nonhost plants.

## Author contributions

X.Z., N.W., P.C.B. and F.B. designed the experiments; X.Z., N.W., C.H., P.C.B. and H.M. performed the experiments; and X.Z., N.W., P.C.B., P.R.J.B., C.H. and F.B. wrote the manuscript.

## Supporting information

Please note: Wiley Blackwell are not responsible for the content or functionality of any Supporting Information supplied by the authors. Any queries (other than missing material) should be directed to the *New Phytologist* Central Office.

**Fig. S1** Alignment of tomato and Arabidopsis CaMs.
**Fig. S2** *In vitro* interaction between C‐terminal 17‐aa domain of SFI5 and His‐SlCaM6.
**Fig. S3** The mutation KR/EE does not abolish GFP‐SFI5 ED plasma membrane localization in *Nicotiana benthamiana* leaves.
**Fig. S4** The CaM binding motif of SFI5 is required for plasma membrane localization in tomato protoplasts.
**Fig. S5** mRFP‐SlCaM3/4/5 and GFP‐SFI5 do not co‐localize in *Nicotiana benthamiana* leaves.
**Fig. S6** Expression of HA‐tagged SFI5 deletion and site‐directed mutants in tomato protoplasts.
**Table S1** List of primers
**Table S2** Potential SFI5‐interacting proteins identified by LC‐MS/MS analysis

## References

[nph15250-bib-0001] Apel K , Hirt H . 2004 Reactive oxygen species: metabolism, oxidative stress, and signal transduction. Annual Review of Plant Biology 55: 373–399. 10.1146/annurev.arplant.55.031903.141701 15377225

[nph15250-bib-0002] Arndt C , Koristka S , Bartsch H , Bachmann M . 2012 Native polyacrylamide gels. Methods in Molecular Biology 869: 49–53. 22585476 10.1007/978-1-61779-821-4_5

[nph15250-bib-0003] Ausubel FM . 2005 Are innate immune signalling pathways in plants and animals conserved? Nature Immunology 6: 973–979. 16177805 10.1038/ni1253

[nph15250-bib-0004] Bhat RA , Miklis M , Schmelzer E , Schulze‐Lefert P , Panstruga R . 2005 Recruitment and interaction dynamics of plant penetration resistance components in a plasma membrane microdomain. Proceedings of the National Academy of Sciences, USA 102: 3135–3140. 10.1073/pnas.0500012102 PMC549507 15703292

[nph15250-bib-0005] Bigeard J , Colcombet J , Hirt H . 2015 Signalling mechanisms in pattern‐triggered immunity (PTI). Molecular Plant 8: 521–539. 25744358 10.1016/j.molp.2014.12.022

[nph15250-bib-0006] Blakesley RW , Boezi JA . 1977 A new staining technique for proteins in polyacrylamide gels using Coomassie brilliant blue G250. Analytical Biochemistry 82: 580–582. 71866 10.1016/0003-2697(77)90197-x

[nph15250-bib-0007] Block A , Alfano JR . 2011 Plant targets for *Pseudomonas syringae* type III effectors: virulence targets or guarded decoys? Current Opinion in Microbiology 14: 39–46. 21227738 10.1016/j.mib.2010.12.011 PMC3040236

[nph15250-bib-0008] Boevink PC , Wang X , McLellan H , He Q , Naqvi S , Armstrong MR , Zhang W , Hein I , Gilroy EM , Tian Z *et al* 2016 A *Phytophthora infestans* RXLR effector targets plant PP1c isoforms that promote late blight disease. Nature Communications 7: 10311. 10.1038/ncomms10311 PMC4740116 26822079

[nph15250-bib-0009] Boller T , Felix G . 2009 A renaissance of elicitors: perception of microbe‐associated molecular patterns and danger signals by pattern‐recognition receptors. Annual Review of Plant Biology 60: 379–406. 10.1146/annurev.arplant.57.032905.105346 19400727

[nph15250-bib-0010] Boller T , He SY . 2009 Innate immunity in plants: an arms race between pattern recognition receptors in plants and effectors in microbial pathogens. Science 324: 742–744. 19423812 10.1126/science.1171647 PMC2729760

[nph15250-bib-0011] Bos JI , Armstrong MR , Gilroy EM , Boevink PC , Hein I , Taylor RM , Zhendong T , Engelhardt S , Vetukuri RR , Harrower B *et al* 2010 *Phytophthora infestans* effector AVR3a is essential for virulence and manipulates plant immunity by stabilizing host E3 ligase CMPG1. Proceedings of the National Academy of Sciences, USA 107: 9909–9914. 10.1073/pnas.0914408107 PMC2906857 20457921

[nph15250-bib-0012] Bos JI , Kanneganti TD , Young C , Cakir C , Huitema E , Win J , Armstrong MR , Birch PR , Kamoun S . 2006 The C‐terminal half of *Phytophthora infestans* RXLR effector AVR3a is sufficient to trigger R3a‐mediated hypersensitivity and suppress INF1‐induced cell death in *Nicotiana benthamiana* . Plant Journal 48: 165–176. 16965554 10.1111/j.1365-313X.2006.02866.x

[nph15250-bib-0013] Bouche N , Yellin A , Snedden WA , Fromm H . 2005 Plant‐specific calmodulin‐binding proteins. Annual Review of Plant Biology 56: 435–466. 10.1146/annurev.arplant.56.032604.144224 15862103

[nph15250-bib-0014] Bozkurt TO , Schornack S , Banfield MJ , Kamoun S . 2012 Oomycetes, effectors, and all that jazz. Current Opinion in Plant Biology 15: 483–492. 22483402 10.1016/j.pbi.2012.03.008

[nph15250-bib-0015] Caillaud MC , Asai S , Rallapalli G , Piquerez S , Fabro G , Jones JD . 2013 A downy mildew effector attenuates salicylic acid‐triggered immunity in Arabidopsis by interacting with the host mediator complex. PLoS Biology 11: e1001732. 24339748 10.1371/journal.pbio.1001732 PMC3858237

[nph15250-bib-0016] Campe R , Langenbach C , Leissing F , Popescu GV , Popescu SC , Goellner K , Beckers GJ , Conrath U . 2016 ABC transporter PEN3/PDR8/ABCG36 interacts with calmodulin that, like PEN3, is required for Arabidopsis nonhost resistance. New Phytologist 209: 294–306. 26315018 10.1111/nph.13582

[nph15250-bib-0017] Chenna R , Sugawara H , Koike T , Lopez R , Gibson TJ , Higgins DG , Thompson JD . 2003 Multiple sequence alignment with the Clustal series of programs. Nucleic Acids Research 31: 3497–3500. 12824352 10.1093/nar/gkg500 PMC168907

[nph15250-bib-0018] Cheval C , Aldon D , Galaud JP , Ranty B . 2013 Calcium/calmodulin‐mediated regulation of plant immunity. Biochimica et Biophysica Acta 1833: 1766–1771. 23380707 10.1016/j.bbamcr.2013.01.031

[nph15250-bib-0019] Chinpongpanich A , Wutipraditkul N , Thairat S , Buaboocha T . 2011 Biophysical characterization of calmodulin and calmodulin‐like proteins from rice, *Oryza sativa* L. Acta Biochimica et Biophysica Sinica 43: 867–876. 21908855 10.1093/abbs/gmr081

[nph15250-bib-0020] Cho KM , Nguyen HT , Kim SY , Shin JS , Cho DH , Hong SB , Shin JS , Ok SH . 2016 CML10, a variant of calmodulin, modulates ascorbic acid synthesis. New Phytologist 209: 664–678. 26315131 10.1111/nph.13612

[nph15250-bib-0021] Crivici A , Ikura M . 1995 Molecular and structural basis of target recognition by calmodulin. Annual Review of Biophysics and Biomolecular Structure 24: 85–116. 10.1146/annurev.bb.24.060195.000505 7663132

[nph15250-bib-0022] Day IS , Reddy VS , Shad Ali G , Reddy AS . 2002 Analysis of EF‐hand‐containing proteins in Arabidopsis. Genome Biology 3: RESEARCH0056. 12372144 10.1186/gb-2002-3-10-research0056 PMC134623

[nph15250-bib-0023] Doehlemann G , Requena N , Schaefer P , Brunner F , O'Connell R , Parker JE . 2014 Reprogramming of plant cells by filamentous plant‐colonizing microbes. New Phytologist 204: 803–814. 25539003 10.1111/nph.12938

[nph15250-bib-0024] Dong S , Yin W , Kong G , Yang X , Qutob D , Chen Q , Kale SD , Sui Y , Zhang Z , Dou D *et al* 2011 *Phytophthora sojae* avirulence effector Avr3b is a secreted NADH and ADP‐ribose pyrophosphorylase that modulates plant immunity. PLoS Pathogens 7: e1002353. 22102810 10.1371/journal.ppat.1002353 PMC3213090

[nph15250-bib-0025] Dou D , Kale SD , Wang X , Jiang RH , Bruce NA , Arredondo FD , Zhang X , Tyler BM . 2008 RXLR‐mediated entry of *Phytophthora sojae* effector Avr1b into soybean cells does not require pathogen‐encoded machinery. Plant Cell 20: 1930–1947. 18621946 10.1105/tpc.107.056093 PMC2518231

[nph15250-bib-0026] Dou D , Zhou JM . 2012 Phytopathogen effectors subverting host immunity: different foes, similar battleground. Cell Host & Microbe 12: 484–495. 23084917 10.1016/j.chom.2012.09.003

[nph15250-bib-0027] Du L , Ali GS , Simons KA , Hou J , Yang T , Reddy AS , Poovaiah BW . 2009 Ca^2+^/calmodulin regulates salicylic‐acid‐mediated plant immunity. Nature 457: 1154–1158. 19122675 10.1038/nature07612

[nph15250-bib-0028] Du Y , Berg J , Govers F , Bouwmeester K . 2015 Immune activation mediated by the late blight resistance protein R1 requires nuclear localization of R1 and the effector AVR1. New Phytologist 207: 735–747. 25760731 10.1111/nph.13355

[nph15250-bib-0029] Fraiture M , Brunner F . 2014 Killing two birds with one stone: trans‐kingdom suppression of PAMP/MAMP‐induced immunity by T3E from enteropathogenic bacteria. Frontiers in Microbiology 5: 320. 25101059 10.3389/fmicb.2014.00320 PMC4105635

[nph15250-bib-0030] Fraiture M , Zheng X , Brunner F . 2014 An Arabidopsis and tomato mesophyll protoplast system for fast identification of early MAMP‐triggered immunity‐suppressing effectors. Methods in Molecular Biology 1127: 213–230. 24643564 10.1007/978-1-62703-986-4_17

[nph15250-bib-0031] Galon Y , Nave R , Boyce JM , Nachmias D , Knight MR , Fromm H . 2008 Calmodulin‐binding transcription activator (CAMTA) 3 mediates biotic defense responses in Arabidopsis. FEBS Letters 582: 943–948. 18298954 10.1016/j.febslet.2008.02.037

[nph15250-bib-0032] Gilroy EM , Taylor RM , Hein I , Boevink P , Sadanandom A , Birch PR . 2011 CMPG1‐dependent cell death follows perception of diverse pathogen elicitors at the host plasma membrane and is suppressed by *Phytophthora infestans* RXLR effector AVR3a. New Phytologist 190: 653–666. 21348873 10.1111/j.1469-8137.2011.03643.x

[nph15250-bib-0033] Giraldo MC , Valent B . 2013 Filamentous plant pathogen effectors in action. Nature Reviews Microbiology 11: 800–814. 24129511 10.1038/nrmicro3119

[nph15250-bib-0034] Guo M , Kim P , Li G , Elowsky CG , Alfano JR . 2016 A bacterial effector co‐opts calmodulin to target the plant microtubule network. Cell Host & Microbe 19: 67–78. 26764598 10.1016/j.chom.2015.12.007

[nph15250-bib-0035] Haas BJ , Kamoun S , Zody MC , Jiang RH , Handsaker RE , Cano LM , Grabherr M , Kodira CD , Raffaele S , Torto‐Alalibo T *et al* 2009 Genome sequence and analysis of the Irish potato famine pathogen *Phytophthora infestans* . Nature 461: 393–398. 19741609 10.1038/nature08358

[nph15250-bib-0500] Halter T , Imkampe J , Mazzotta S , Wierzba M , Postel S , Bucherl C , Kiefer C , Stahl M , Chinchilla D , Wang X *et al* 2014 The leucine‐rich repeat receptor kinase BIR2 is a negative regulator of BAK1 in plant immunity. Current Biology 24: 134‐143. 24388849 10.1016/j.cub.2013.11.047

[nph15250-bib-0036] He P , Shan L , Lin NC , Martin GB , Kemmerling B , Nürnberger T , Sheen J . 2006 Specific bacterial suppressors of MAMP signalling upstream of MAPKKK in Arabidopsis innate immunity. Cell 125: 563–575. 16678099 10.1016/j.cell.2006.02.047

[nph15250-bib-0037] Jiang RH , Tripathy S , Govers F , Tyler BM . 2008 RXLR effector reservoir in two *Phytophthora* species is dominated by a single rapidly evolving superfamily with more than 700 members. Proceedings of the National Academy of Sciences, USA 105: 4874–4879. 10.1073/pnas.0709303105 PMC2290801 18344324

[nph15250-bib-0038] Karimi M , De Meyer B , Hilson P . 2005 Modular cloning and expression of tagged fluorescent protein in plant cells. Trends in Plant Sciences 10: 103–105. 10.1016/j.tplants.2005.01.008 15749466

[nph15250-bib-0040] Kim MC , Panstruga R , Elliott C , Muller J , Devoto A , Yoon HW , Park HC , Cho MJ , Schulze‐Lefert P . 2002 Calmodulin interacts with MLO protein to regulate defence against mildew in barley. Nature 416: 447–451. 11919636 10.1038/416447a

[nph15250-bib-0041] King SR , McLellan H , Boevink PC , Armstrong MR , Bukharova T , Sukarta O , Win J , Kamoun S , Birch PR , Banfield MJ . 2014 *Phytophthora infestans* RXLR effector PexRD2 interacts with host MAPKKK epsilon to suppress plant immune signalling. Plant Cell 26: 1345–1359. 24632534 10.1105/tpc.113.120055 PMC4001388

[nph15250-bib-0042] Kong L , Qiu X , Kang J , Wang Y , Chen H , Huang J , Qiu M , Zhao Y , Kong G , Ma Z . 2017 A *Phytophthora* effector manipulates host histone acetylation and reprograms defense gene expression to promote infection. Current Biology 27: 981–991. 28318979 10.1016/j.cub.2017.02.044

[nph15250-bib-0043] Kong G , Zhao Y , Jing M , Huang J , Yang J , Xia Y , Kong L , Ye W , Xiong Q , Qiao Y *et al* 2015 The activation of *Phytophthora* effector Avr3b by plant cyclophilin is required for the nudix hydrolase activity of Avr3b. PLoS Pathogens 11: e1005139. 26317500 10.1371/journal.ppat.1005139 PMC4552650

[nph15250-bib-0044] Kurup S , Runions J , Kohler U , Laplaze L , Hodge S , Haseloff J . 2005 Marking cell lineages in living tissues. Plant Journal 42: 444–453. 15842628 10.1111/j.1365-313X.2005.02386.x

[nph15250-bib-0045] Lecourieux D , Ranjeva R , Pugin A . 2006 Calcium in plant defence‐signalling pathways. New Phytologist 171: 249–269. 16866934 10.1111/j.1469-8137.2006.01777.x

[nph15250-bib-0046] Lee J , Eschen‐Lippold L , Lassowskat I , Bottcher C , Scheel D . 2015 Cellular reprogramming through mitogen‐activated protein kinases. Frontiers in Plant Science 6: 940. 26579181 10.3389/fpls.2015.00940 PMC4625042

[nph15250-bib-0047] Macho AP , Zipfel C . 2014 Plant PRRs and the activation of innate immune signalling. Molecular Cell 54: 263–272. 24766890 10.1016/j.molcel.2014.03.028

[nph15250-bib-0048] McCormack E , Tsai YC , Braam J . 2005 Handling calcium signalling: Arabidopsis CaMs and CMLs. Trends in Plant Science 10: 383–389. 16023399 10.1016/j.tplants.2005.07.001

[nph15250-bib-0049] McLellan H , Boevink PC , Armstrong MR , Pritchard L , Gomez S , Morales J , Whisson SC , Beynon JL , Birch PR . 2013 An RxLR effector from *Phytophthora infestans* prevents re‐localisation of two plant NAC transcription factors from the endoplasmic reticulum to the nucleus. PLoS Pathogens 9: e1003670. 24130484 10.1371/journal.ppat.1003670 PMC3795001

[nph15250-bib-0050] Meador WE , Means AR , Quiocho FA . 1992 Target enzyme recognition by calmodulin: 2.4 A structure of a calmodulin‐peptide complex. Science 257: 1251–1255. 1519061 10.1126/science.1519061

[nph15250-bib-0051] Medzhitov R , Janeway CA Jr . 1997 Innate immunity: the virtues of a nonclonal system of recognition. Cell 91: 295–298. 9363937 10.1016/s0092-8674(00)80412-2

[nph15250-bib-0052] Mruk K , Farley BM , Ritacco AW , Kobertz WR . 2014 Calmodulation meta‐analysis: predicting calmodulin binding via canonical motif clustering. Journal of General Physiology 144: 105–114. 24935744 10.1085/jgp.201311140 PMC4076516

[nph15250-bib-0053] Niepmann M , Zheng J . 2006 Discontinuous native protein gel electrophoresis. Electrophoresis 27: 3949–3951. 16991206 10.1002/elps.200600172

[nph15250-bib-0054] Nürnberger T , Brunner F . 2002 Innate immunity in plants and animals: emerging parallels between the recognition of general elicitors and pathogen‐associated molecular patterns. Current Opinion in Plant Biology 5: 318–324. 12179965 10.1016/s1369-5266(02)00265-0

[nph15250-bib-0055] Nürnberger T , Brunner F , Kemmerling B , Piater L . 2004 Innate immunity in plants and animals: striking similarities and obvious differences. Immunological Reviews 198: 249–266. 15199967 10.1111/j.0105-2896.2004.0119.x

[nph15250-bib-0056] O'Neil KT , DeGrado WF . 1990 How calmodulin binds its targets: sequence independent recognition of amphiphilic α‐helices. Trends in Biochemical Sciences 15: 59–64. 2186516 10.1016/0968-0004(90)90177-d

[nph15250-bib-0057] Pieterse CMJ , Davidse LC . 1990 Attempted cloning of the *Phytophthora infestans* pyr‐gene for use in a homologous transformation system. Phytophthora Newsletter 16: 7–8.

[nph15250-bib-0058] Reddy AS , Ali GS , Celesnik H , Day IS . 2011 Coping with stresses: roles of calcium‐ and calcium/calmodulin‐regulated gene expression. Plant Cell 23: 2010–2032. 21642548 10.1105/tpc.111.084988 PMC3159525

[nph15250-bib-0059] Shan L , Thara VK , Martin GB , Zhou JM , Tang X . 2000 The pseudomonas AvrPto protein is differentially recognized by tomato and tobacco and is localized to the plant plasma membrane. Plant Cell 12: 2323–2338. 11148281 10.1105/tpc.12.12.2323 PMC102221

[nph15250-bib-0060] Tyler BM , Tripathy S , Zhang X , Dehal P , Jiang RH , Aerts A , Arredondo FD , Baxter L , Bensasson D , Beynon JL *et al* 2006 *Phytophthora* genome sequences uncover evolutionary origins and mechanisms of pathogenesis. Science 313: 1261–1266. 16946064 10.1126/science.1128796

[nph15250-bib-0061] Underwood W , Somerville SC . 2013 Perception of conserved pathogen elicitors at the plasma membrane leads to relocalization of the Arabidopsis PEN3 transporter. Proceedings of the National Academy of Sciences, USA 110: 12492–12497. 10.1073/pnas.1218701110 PMC3725069 23836668

[nph15250-bib-0062] Whisson SC , Boevink PC , Moleleki L , Avrova AO , Morales JG , Gilroy EM , Armstrong MR , Grouffaud S , van West P , Chapman S *et al* 2007 A translocation signal for delivery of oomycete effector proteins into host plant cells. Nature 450: 115–118. 17914356 10.1038/nature06203

[nph15250-bib-0063] Whisson SC , Boevink PC , Wang S , Birch PRJ . 2016 The cell biology of late blight disease. Current Opinion in Microbiology 34: 127–135. 27723513 10.1016/j.mib.2016.09.002 PMC5340842

[nph15250-bib-0064] Xin XF , He SY . 2013 *Pseudomonas syringae* pv. tomato DC3000: a model pathogen for probing disease susceptibility and hormone signalling in plants. Annual review of Phytopathology 51: 473–498. 10.1146/annurev-phyto-082712-102321 23725467

[nph15250-bib-0066] Yang L , McLellan H , Naqvi S , He Q , Boevink PC , Armstrong M , Giuliani LM , Zhang W , Tian Z , Zhan J *et al* 2016 Potato NPH3/RPT2‐like protein StNRL1, targeted by a *Phytophthora infestans* RXLR effector, is a susceptibility factor. Plant Physiology 171: 645–657. 26966171 10.1104/pp.16.00178 PMC4854710

[nph15250-bib-0067] Yap KL , Kim J , Truong K , Sherman M , Yuan T , Ikura M . 2000 Calmodulin target database. Journal of Structural and Functional Genomics 1: 8–14. 12836676 10.1023/a:1011320027914

[nph15250-bib-0068] Zhang W , Zhou RG , Gao YJ , Zheng SZ , Xu P , Zhang SQ , Sun DY . 2009 Molecular and genetic evidence for the key role of AtCaM3 in heat‐shock signal transduction in Arabidopsis. Plant Physiology 149: 1773–1784. 19211698 10.1104/pp.108.133744 PMC2663753

[nph15250-bib-0069] Zhao Y , Liu W , Xu YP , Cao JY , Braam J , Cai XZ . 2013 Genome‐wide identification and functional analyses of calmodulin genes in *Solanaceous* species. BMC Plant Biology 13: 70. 23621884 10.1186/1471-2229-13-70 PMC3751459

[nph15250-bib-0070] Zheng X , McLellan H , Fraiture M , Liu X , Boevink PC , Gilroy EM , Chen Y , Kandel K , Sessa G , Birch PR *et al* 2014 Functionally redundant RXLR effectors from *Phytophthora infestans* act at different steps to suppress early flg22‐triggered immunity. PLoS Pathogens 10: e1004057. 24763622 10.1371/journal.ppat.1004057 PMC3999189

