# Supplementary material for: Phytophthora infestans RXLR effector SFI5 requires association with calmodulin for PTI/MTI suppressing activity
Source: New Phytol. 2018 Jun 22;219(4):1433–46. doi: 10.1111/nph.15250 (PMC6099356; doi:10.1111/nph.15250)
Supplement: Supplementary file 1 — Fig. S1 Alignment of tomato and Arabidopsis CaMs. Fig. S2 In vitro interaction between C‐terminal 17‐aa domain of SFI5 and His‐SlCaM6. Fig. S3 The mutation KR/EE does not abolish GFP‐SFI5 ED plasma membrane localization in Nicotiana benthamiana leaves. Fig. S4 The CaM binding motif of SFI5 is required for plasma membrane localization in tomato protoplasts. Fig. S5 mRFP‐SlCaM3/4/5 and GFP‐SFI5 do not co‐localize in Nicotiana benthamiana leaves. Fig. S6 Expression of HA‐tagged SFI5 deletion and site‐directed mutants in tomato protoplasts. Table S1 List of primers Table S2 Potential SFI5‐interacting proteins identified by LC‐MS/MS analysis [file NPH-219-1433-s001.pdf]

***New Phytologist* Supporting Information**

Article title: *Phytophthora infestans* RXLR effector SFI5 requires association with Calmodulin for PTI/MTI suppressing activity

Authors: Xiangzi Zheng, Nadine Wagener, Hazel McLellan, Petra C Boevink, Chenlei Hua, Paul R.J. Birch, Frédéric Brunner

Article acceptance date: 03 May 2018

The following Supporting Information is available for this article:

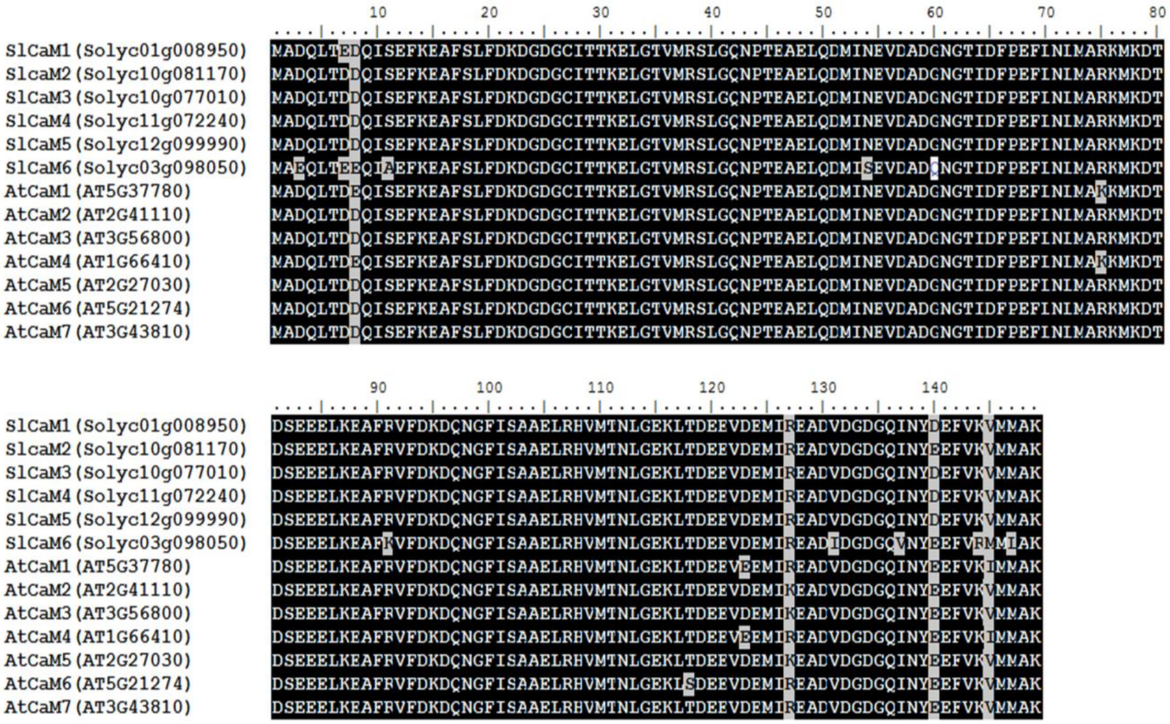

Fig. S1 Alignment of Tomato and Arabidopsis CaMs. The full-length protein sequences of CaMs were aligned by ClustalW. The accession numbers for these CaMs are indicated in the brackets.

(a)

Peptide No.	Sequence	pI	Net charge
1	S T W K I F K I I S R L K K L K L K R	12.137	7.903
2	S T W K I F K I I S R L K K L K L	11.434	5.904
6	S T W K I F E I I S R L E E L K L K R	9.706	1.914
10	S T A K I A K I I S R L K K L K L	11.434	5.907
11	S T A K I A K I I S R L E E L K L K R	10.453	3.910
12	S T H K I H K I I S R L K K L K L K R	12.140	8.240
13	S T E K I E K I I S R L K K L K L K R	10.660	5.910
14	S T E K I E K I I S R L E E L K L K R	9.540	1.920

(b)

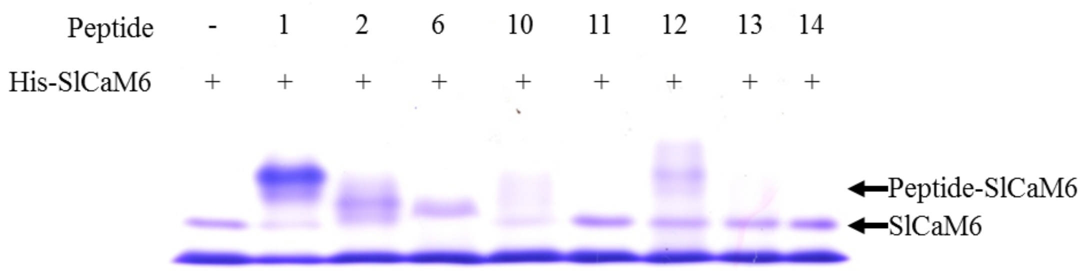

Fig. S2 In vitro interaction between C-terminal 17-aa domain of SFI5 and His-SlCaM6
(a) Synthetic peptides corresponding to the predicted CaM-binding site with various amino acid mutations. Peptide 1 represents the last 19aa at the C-terminus of SFI5. Peptides 2, 6, 10-14 are truncated or mutated versions of peptide 1, in which the substituted amino acids are presented in red. The pI and net charge (at pH7.0) of each peptide were calculated by Editseq (Lasergene v.8; DNASTAR). (b) CaM mobility shift on tris/glycine native gels. Purified His-SlCaM6 (50 µM) was incubated with each synthetic peptide (133 µM) in the presence of 5 mM $CaCl_2$. Samples were separated on tris/glycine native gels followed by Coomasssie blue staining. Arrows indicates the position of bands representing free His-SlCaM6 and peptide-His-SlCaM6 complex.

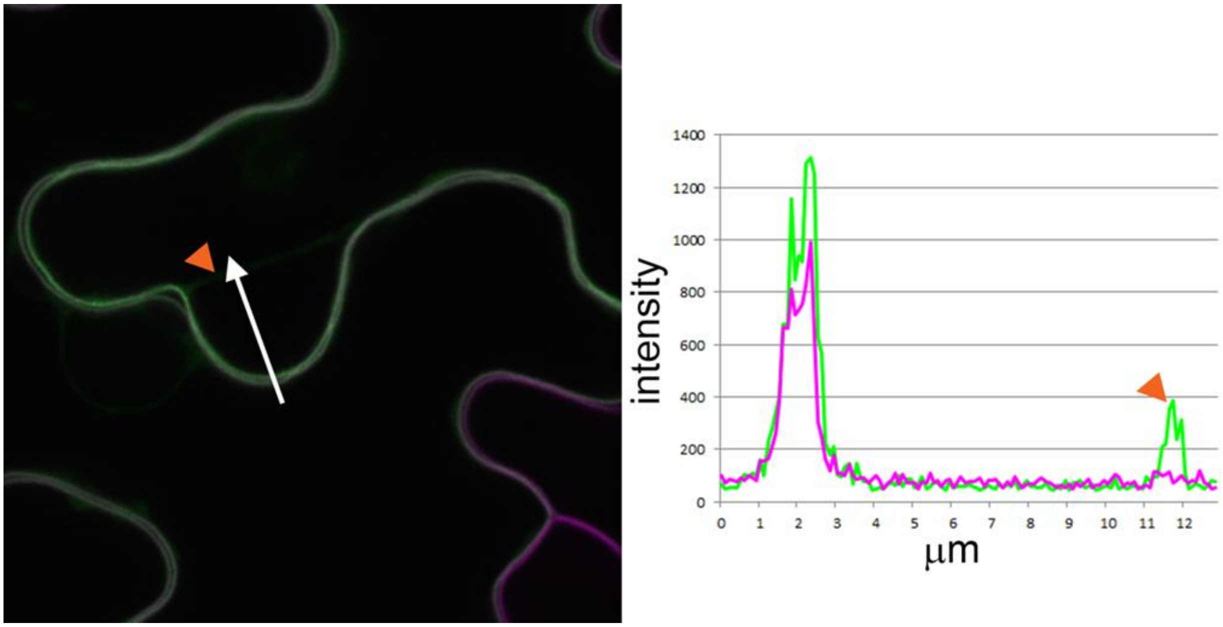

Fig. S3 The mutation KR/EE does not abolish GFP-SFI5 ED plasma membrane localization in *N. benthamiana* leaves. An example single optical section image of the GFP-SFI5 ED KR/EE mutant (green) co-expressed with the PM marker mOrange-LTi6b (magenta). The arrow indicates the line drawn to generate the intensity profile shown to the right of the image. The profile was drawn such that a cytoplasmic strand (indicated by the orange arrowhead in the image and profile) and the plasma membrane were crossed. The length of the profile is shown in the graph in mm and the intensities are as generated by the confocal software.

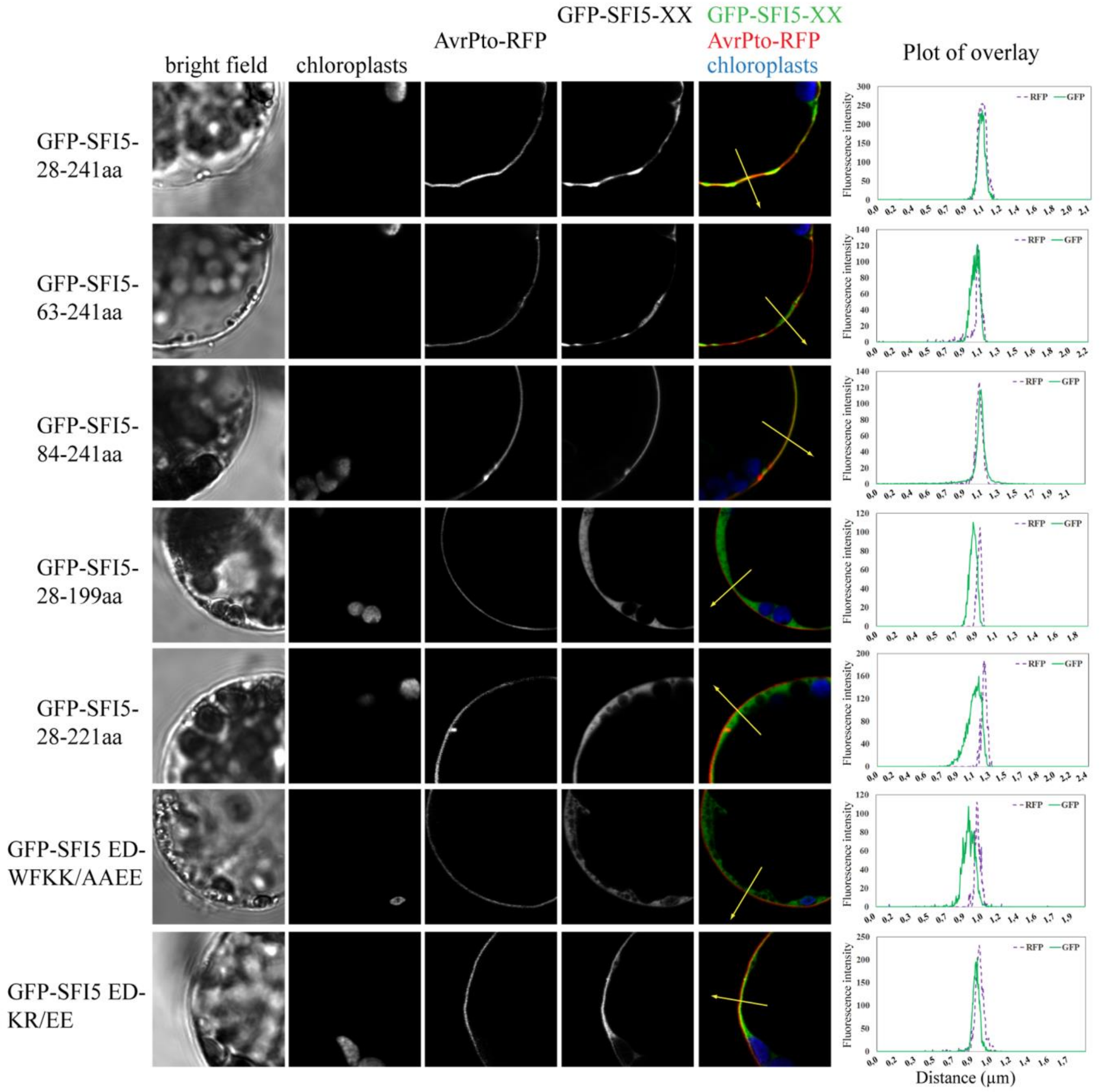

Fig. S4 The CaM binding motif of SFI5 is required for plasma membrane localization in tomato protoplasts. Tomato protoplasts co-expressing N-terminally GFP-tagged SFI5 deletion or point mutants (as indicated to the left of each image row) and C-terminally RFP-tagged AvrPto were imaged using confocal microscopy. Images were taken 12 hours after transfection and each line of images shows optical sections of bright field, chloroplast, RFP, GFP and merged fluorescence as indicated. ImageJ software was used to analyze the signal intensity of GFP and RFP fluorescence in the overlay picture along the lines indicated by the yellow arrows (each 0.1 µm in length). Imaging was performed using Leica TCS SP8 AOBS confocal microscopes with HC PL APO 63×1.20W water immersion objectives. Samples were excited and emitted by an argon/krypton mixed gas laser. The following settings were used for: GFP, 488nm/496-520nm; mRFP, 561nm/575-605nm and chloroplasts, 633nm/647-685nm. The pinhole was adjusted to 1.0 airy unit for each wavelength.

(a)

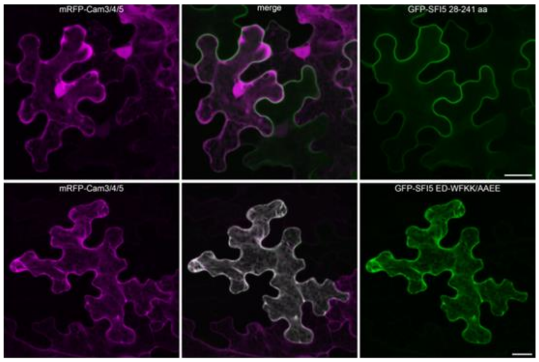

(b)

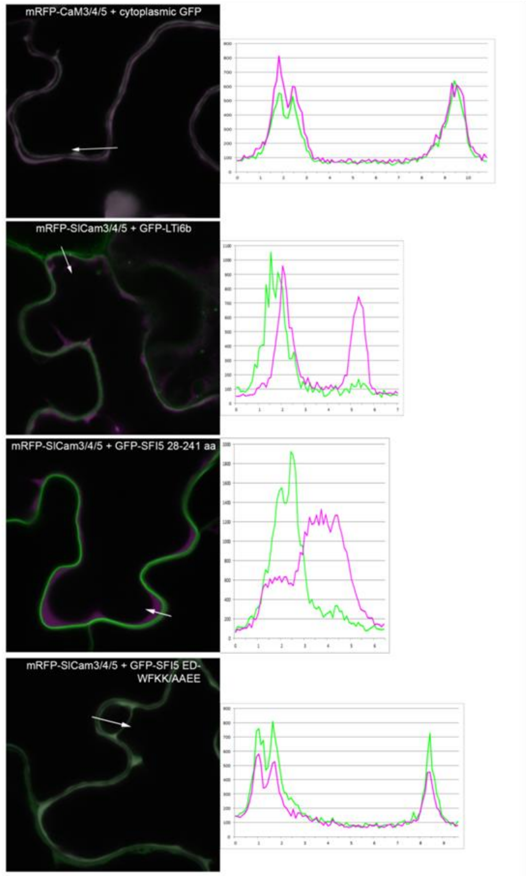

Fig. S5 mRFP-SlCaM3/4/5 and GFP-SFI5 do not co-localize in *N. benthamiana* leaves. (a) Confocal projection images of cells of *N. benthamiana* leaves co-expressing mRFP-SlCaM3/4/5 (magenta) and GFP-SFI5 28-241 aa or GFP-SFI5 ED WFKK/AAEE (in green; as indicated). GFP-SFI5 28-241 aa displays a typical plasma membrane localisation pattern. mRFP-SlCaM3/4/5 and GFP-SFI5 ED WFKK/AAEE display typical cytoplasmic labelling. Scale bars represent 20 mm. (b) Single optical section images of mRFP-SlCaM3/4/5 co-expressed with cytoplasmic GFP, the PM marker GFP-LTi6b, GFP-SFI5 28-241 aa or GFP-SFI5 ED WFKK/AAEE (as indicated). The arrows indicate the lines drawn to generate the intensity profiles shown to the right of each image. Each profile was drawn such that a cytoplasmic strand, or region of cytoplasm, and the plasma membrane were crossed. The lengths of the profiles are shown in the graphs in mm and the intensities are as generated by the confocal software.

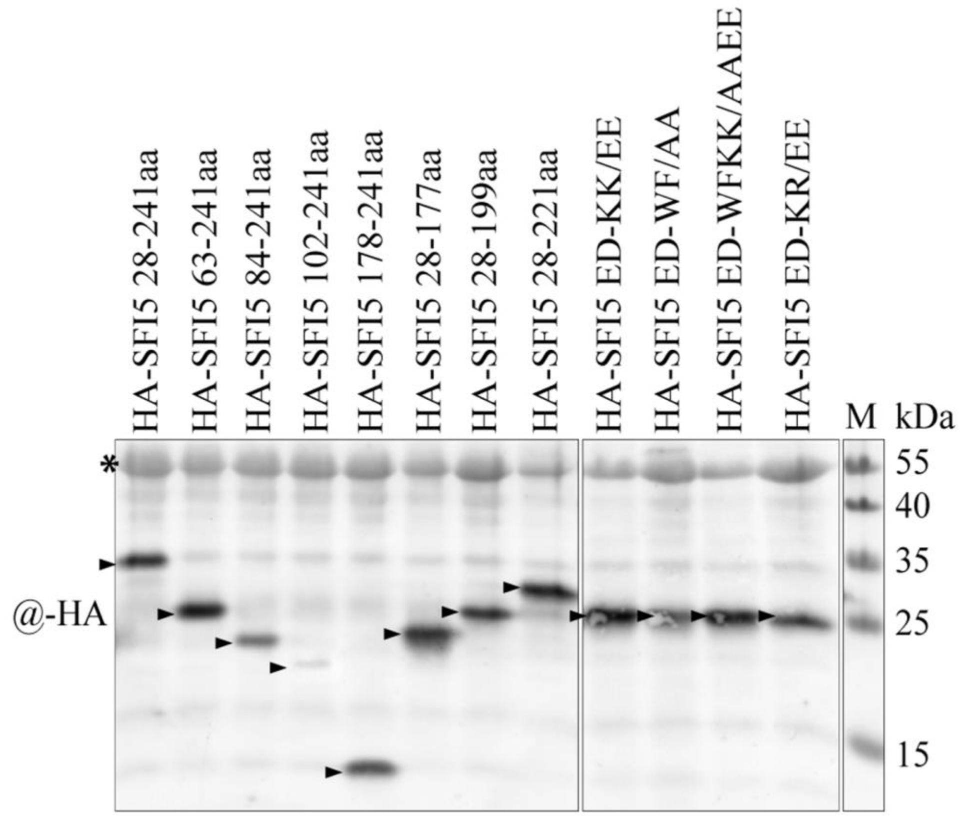

Fig. S6 Expression of HA-tagged SFI5 deletion and site-directed mutants in tomato protoplasts. Immunodetection of HA-SFI deletion and point mutants transiently expressed in tomato protoplasts for 24 h was performed with anti-HA antibody. Signals corresponding to the different HA fusion proteins are pointed out with an arrow. The asterisk indicates the position of the non-specific Rubisco protein, serving as a loading control. The experiment is representative of three repeats.

Table S1 List of used primers

Primers used for the cloning of SlCaMs	5'-3' sequence
SlCaM1-attB1	AAAAAGCAGGCTTCATGGCGGATCAGCTCACCGAA
SlCaM1-attB2	AGAAAGCTGGGTCTCACTTAGCCATCATAACCTTGAC
SlCaM3/4/5-attB1	AAAAAGCAGGCTTCATGGCGGATCAGCTTACAGATG
SlCaM3/4/5-attB2	AGAAAGCTGGGTCTCACTTAGCCATCATGAC
SlCaM6-attB1	AAAAAGCAGGCTTCATGGCAGAGCAGCTGAC
SlCaM6-attB2	AGAAAGCTGGGTCTCACTTGGCAAGCATCAT
attB1-adapter	GGGGACAAGTTTGTACAAAAAAGCAGGCT
attB2-adapter	GGGGACCACTTTGTACAAGAAAGCTGGGT
Primers used for generating the SFI5 deletion constructs and site-directed mutagenesis (mutated nucleotides are underlined)	
SFI5 28aa-attB1	AAAAAGCAGGCTTCACCATGGCCTCCGACCAGAAT
SFI5 63aa-attB1	AAAAAGCAGGCTTCACCATGTTCCTGACAGAACCCCC
SFI5 84aa-attB1	AAAAAGCAGGCTTCACCATGAACAGCGGGCTACCAGAT
SFI5 102aa-attB1	AAAAAGCAGGCTTCACCATGAACAGCGGGCTACCAGAT
SFI5 178aa-attB1	AAAAAGCAGGCTTCACCATGGATCCTTTAAATAGGGAGCAG
SFI5 177aa-attB2	AGAAAGCTGGGTCTTAAAGCTTGACAATGGCCGA
SFI5 199aa-attB2	AGAAAGCTGGGTCTTAAGCCTGATTCTTTTTAAGAGCAA
SFI5 221aa-attB2	AGAAAGCTGGGTCTTATTTGGACTTGGCTGCCATA
SFI5 241aa-attB2	AGAAAGCTGGGTCTCAACGTTTTAGCTTTAATTTTT
SFI5 ED-WF/AA-1	AGCACGGCTAAAATCGCTAAAATTATCTCGA
SFI5 ED-WF/AA-2	TTAGCGATTTTAGCCGTGCTTGGTTTGGA
SFI5 ED-KK/EE-1	GGCTTGAGGAGTTAAAGCTAAAACGTTAACACCC
SFI5 ED-KK/EE-2	TTAACTCCTCAAGCCTCGAGATAATTTTAAAGATTTTC
SFI5 ED-KR/EE-1	GCTAGAGGAGTAACACCCAGCTTTCTTGTAC
SFI5 ED-KR/EE-2	TGTTACTCCTCTAGCTTTAATTTTTTAAGCCTCG
XmnI-SFI5-F	CCGGAAGGATTTCATGGCCTCCGACCAGAAT
SalI-SFI5-R	ACGCGTCGACTCAACGTTTTAGCTTTAATTTTT
SalI-SFI5-R	ACGCGTCGACTCAACGTTTTAGCTTTAATTTTT

Table S2 Potential SFI5-interacting proteins identified by LC-MS/MS analysis. Total protein extracts from tomato protoplasts expressing HA-SFI5 were immunoprecipitated with anti-HA affinity matrix and the eluted proteins were subject to liquid chromatography followed by LC-MS/MS. Protoplasts expressing the empty vector or HA-SFI1 (Zheng et al., 2014) served as control for interactor specificity. The table is listing putative protein interacting candidates that were identified only in the presence of HA-SFI5.

		Experiment (HA-SFI5)	
**Protein ID**	**Protein Descriptions**	**Intensity**	**# of Peptide**
Q8RXB8_SOLLC	N-hydroxycinnamoyl-CoA:tyramine N-hydroxycinnamoyl transferase THT1-3; [Solanum lycopersicum (Tomato) (Lycopersicon esculentum).]	4588200	1
IPI00938824.1	CAM1 (CALMODULIN 1); calcium ion binding;Calmodulin-1/4	2128200	3
IPI00548063.1	60S ribosomal protein L5-1;60S ribosomal protein L5-2	1567500	1
IPI00543640.1	50S ribosomal protein L17, chloroplastic	1428200	1
IPI00548475.1	60S ribosomal protein L18a-2	1195800	2
IPI00536958.1	Calmodulin-7;Calmodulin-2/3/5;Calmodulin-6;CAM5 (CALMODULIN 5)	959950	3
IPI00657454.1	EIF4A1 (EUKARYOTIC TRANSLATION INITIATION FACTOR 4A1); ATP-dependent helicase/ translation initiation	572990	2
RR8_SOLLC	30S ribosomal protein S8, chloroplastic; [Solanum lycopersicum (Tomato) (Lycopersicon esculentum).]	552660	3
IPI00526224.1	30S ribosomal protein S18, chloroplastic; [Solanum lycopersicum (Tomato) (Lycopersicon esculentum).]	528660	3
TL29_SOLLC	Thylakoid lumenal 29 kDa protein, chloroplastic; [Solanum lycopersicum (Tomato) (Lycopersicon esculentum).]	378590	2
IPI00528531.1	Calmodulin-like protein 8	333140	1
IPI00529254.2	30S ribosomal protein S14, chloroplastic; [Solanum lycopersicum (Tomato) (Lycopersicon esculentum).]	318030	1
RR16_SOLLC	30S ribosomal protein S16, chloroplastic; [Solanum lycopersicum (Tomato) (Lycopersicon esculentum).]	298370	1
IPI00539263.1	Prx B 2-Cys Prx B (2-Cysteine peroxiredoxin B); antioxidant/ peroxiredoxin	221600	1
IPI00520130.1	ATP synthase alpha chain, mitochondrial, putative;ATP synthase subunit alpha, mitochondrial;ATPase subunit 1	209090	1
IPI00519748.1	Glutamine synthetase cytosolic isozyme 1-1	177900	1
O49877_SOLLC	Cysteine protease TDI-65; [Solanum lycopersicum (Tomato) (Lycopersicon esculentum).]	152200	1
IPI00520128.1	AtRABA1e (Arabidopsis Rab GTPase homolog A1e);Putative GTP-binding protein	140620	1
IPI00543126.2	Chlorophyll a-b binding protein 7, chloroplastic; [Solanum lycopersicum (Tomato) (Lycopersicon esculentum).]	136110	1
IPI00539779.3	Putative vacuolar proton ATPase subunit E; [Solanum lycopersicum (Tomato) (Lycopersicon esculentum).]	132260	1
Q9M7M2_SOLLC	DnaJ-like protein; [Solanum lycopersicum (Tomato) (Lycopersicon esculentum).]	102830	1
IPI00542662.1	26S protease regulatory subunit 8 homolog A;26S protease regulatory subunit 8 homolog B	63488	1
IPI00539067.1	Probable UDP-glucose 6-dehydrogenase 2	62907	1
Q4W5U8_SOLLC	FtsH protease; [Solanum lycopersicum (Tomato) (Lycopersicon esculentum).]	57105	1
Q9LEG3_SOLLC	Putative alcohol dehydrogenase; [Solanum lycopersicum (Tomato) (Lycopersicon esculentum).]	50268	1
IPI00526133.1	Proteasome subunit alpha type-5-A	47313	1
C6K2K9_SOLLC	GDP-mannose 3',5'-epimerase; EC=5.1.3.18; [Solanum lycopersicum (Tomato) (Lycopersicon esculentum).]	41934	2
A7LI54_SOLLC	Phototropin-2; [Solanum lycopersicum (Tomato) (Lycopersicon esculentum).]	31052	1
IPI00938787.1	phosphoglucomutase, cytoplasmic, putative / glucose phosphomutase, putative	30334	1

Zheng X, McLellan H, Fraiture M, Liu X, Boevink PC, Gilroy EM, Chen Y, Kandel K, Sessa G, Birch PR, Brunner F. 2014. Functionally redundant RXLR effectors from *Phytophthora infestans* act at different steps to suppress early flg22-triggered immunity. *PLoS Pathog* **10**(4): e1004057.
